# Exacerbation Risk by Chronic Proton Pump Inhibitor Use in Obstructive Lung Diseases

**DOI:** 10.1016/j.chest.2026.01.002

**Published:** 2026-01-16

**Authors:** Valerie Dehondt, Frauke Van Vaerenbergh, Katia Verhamme, Lies Lahousse

**Affiliations:** a Pharmaceutical Care Unit, Department of Bioanalysis, Faculty of Pharmaceutical Sciences, Ghent University, Ghent, Belgium; b Department of Medical Informatics, Erasmus University Medical Center, Rotterdam, The Netherlands; c Department of Epidemiology, Erasmus University Medical Center, Rotterdam, The Netherlands

**Keywords:** asthma, chronic obstructive airway diseases, COPD, exacerbations, gastroesophageal reflux disease, proton pump inhibitors

## Abstract

**Background:**

Previous studies have shown inconsistent results regarding the use of proton pump inhibitors (PPIs) and the risk on exacerbations in patients with chronic obstructive airway diseases (COADs).

**Research Question:**

Is long-term PPI use associated with exacerbation risk?

**Study Design and Methods:**

Using Belgian nationwide claims-based data of adult patients receiving long-term medication for COADs between 2017 and 2022, we investigated the association between PPI use and exacerbation risk using multivariable Cox models. Based on defined daily doses (DDDs) dispensed during the prior year, dose-dependent associations were assessed by inverse probability of treatment weighted Cox regression models.

**Results:**

Among 932,135 included patients with COADs, 416,087 patients (44.6%) were PPI users, of whom 57,540 patients (13.8%) received 1-28 DDDs of PPIs, 128,017 patients (30.8%) received 29-180 DDDs, 127,981 patients (30.8%) received 181-365 DDDs, and 102,549 patients (24.6%) received > 365 DDDs in the previous year. Beyond age, sex, smoking, socioeconomic status, exacerbation history, short-acting bronchodilator use, frailty, and comorbidities, PPI use was associated with an increased risk of exacerbations (adjusted hazard ratio [HR], 1.18 [95% CI, 1.17-1.19]). Moreover, compared with patients who did not use PPIs, the risk of exacerbations increased with cumulative DDDs (≤ 28 DDDs: HR, 1.09 [95% CI, 1.07-1.10]; ≤ 180 DDDs: HR, 1.15 [95% CI, 1.14-1.16]; ≤ 365 DDDs: HR, 1.19 [95% CI, 1.18-1.20]; and > 365 DDDs: HR, 1.25 [95% CI, 1.23-1.26]). Sensitivity analyses indicated that the association with exacerbation risk was most pronounced in patients younger than 50 years, who were not frail, and with theoretically increased PPI plasma concentrations, and was not significant when used short-term in patients with gastroesophageal reflux disease.

**Interpretation:**

Cumulative PPI use in patients with obstructive lung diseases was associated with an increase in exacerbation risk. This highlights the need to consider PPI use carefully in clinical respiratory practice.

FOR EDITORIAL COMMENT, SEE PAGE 1157

**Take-Home Points**

**Research Question:** Are proton pump inhibitors (PPIs) associated with an increased exacerbation risk in patients with chronic obstructive airway diseases?

**Results:** PPI use was associated with increased exacerbation risk, with the strongest effect in young patients, patients with asthma (overlap), patients without gastroesophageal reflux disease, and long-term users of PPIs.

**Interpretation:** Our results show that it is essential to reconsider PPI use carefully in clinical practice, especially in patients without an ongoing clear indication.

Chronic obstructive airway diseases (COADs), such as asthma and COPD, are highly prevalent worldwide, bringing a substantial economic and societal burden.^1^^,^^2^ Both airway diseases are characterized by airflow obstruction and chronic inflammation.^1^, ^2^, ^3^ Furthermore, patients may experience lung attacks (exacerbations), which are acute episodes of worsening respiratory symptoms associated with increased local and systemic inflammation.^1^^,^^2^ Exacerbations are associated with accelerated lung function decline, impaired quality of life, and increased mortality risk.^4^, ^5^, ^6^ Comorbidities are frequent in COADs and may affect the disease course.

Gastroesophageal reflux disease (GERD) is a common comorbidity in both COPD and asthma.^7^ GERD is a disorder in which reflux of gastric contents into the oesophagus or mouth causes typical symptoms such as heartburn and regurgitation, complications, or both. Additionally, extraoesophageal symptoms may occur, including chronic cough, wheezing, and sore throat.^8^^,^^9^ A 4- to 8-week course of a proton pump inhibitor (PPI) is recommended as first-line pharmacologic treatment in the management of GERD.^8^^,^^9^ However, PPIs are often overused, with one-half of their use being inappropriate.^10^ This raises concerns about adverse events associated with long-term use.^11^

GERD is associated with worse symptoms and higher exacerbation risk in patients with COADs.^7^^,^^12^^,^^13^ It was hypothesized that using PPIs for GERD might reduce exacerbation risk.^14^ However, previous studies showed inconsistent results regarding PPI use and COAD exacerbations.^7^^,^^14^, ^15^, ^16^, ^17^ It remains unclear whether PPIs prevent exacerbations.^1^ Therefore, we investigated a dose-response association between PPIs and respiratory exacerbations in the largest cohort to date of patients with COADs.

## Study Design and Methods

### Study Population

From January 1, 2017, through December 31, 2021, adult patients with ≥ 2 dispensings of medication for obstructive lung disease (Anatomical Therapeutic Chemical code R03) from a Belgian outpatient pharmacy within 1 year and having at least 1 year of Belgian health insurance coverage were included.^18^ The index date was the delivery date of the second package, which resulted in the patient’s inclusion (e-Fig 1). Patients with contraindications to PPIs (methotrexate use or liver cirrhosis) were excluded.^19^^,^^20^ Additionally, those using histamine type 2 receptor antagonists were excluded, because authorities suspended these in mid 2019 because of concerns about a potential carcinogen impurity.^21^ Follow-up ended on occurrence of the outcome (ie, exacerbations), death, emigration, or end of study, whichever came first. This study was approved by the InterMutualistic Agency and Minimal Hospital Dataset database administrators and the Social Security and Health Chamber of the Belgian Information Security Committee (approval code IVC/KSZG/23/008), waiving the need for individual informed consent.^22^ The study was designed according to the Strengthening the Reporting of Observational Studies in Epidemiology guidelines (e-Table 1),^23^ and methodologic details were published and are provided in e-Appendix 1.^18^^,^^24^

### Exposure

The exposure of interest was the use of PPIs, defined by Anatomical Therapeutic Chemical code A02BC. Low-dose over-the-counter PPIs were not registered. For the dose-response relationship, the defined daily dose (DDD) according to the World Health Organization was used to calculate the cumulative PPI dose based on both inpatient and outpatient prescriptions in the year before the index date. The cumulative PPI dose was calculated and classified into 1-28 DDDs, 29-180 DDDs, 181-365 DDDs, and > 365 DDDs.

### Outcomes

The primary outcome of interest was occurrence of exacerbation, including moderate and severe (requiring hospitalization) exacerbations, with definitions based on Global Initiative for Chronic Obstructive Lung Disease and Global Initiative for Asthma reports.^1^^,^^2^ Moderate exacerbations were defined as an outpatient prescription fill for an oral corticosteroid, guideline-recommended antibiotics, or both.^25^ Prescription fills needed to be separated by at least 14 days to be considered as separate events. Moderate exacerbations that led to hospital admission for a severe exacerbation within 14 days were classified only as exacerbations requiring hospitalization to avoid counting the same event twice. Exacerbations requiring hospitalization were defined as hospital admissions or emergency department visits with a primary diagnosis code of asthma or (acute) COPD exacerbation, COPD with acute lower respiratory infection or asthma with status asthmaticus, or a primary diagnosis of chronic lower respiratory disease or respiratory failure, combined with a secondary diagnosis code for asthma or (acute) COPD exacerbation, COPD with acute lower respiratory infection, or asthma with status asthmaticus. Hospitalizations with a concomitant pneumonia diagnosis were excluded (e-Table 2).^1^^,^^2^ The incident date of an exacerbation was the date of the first prescription fill of treatment for a moderate exacerbation or the hospital admission date in case of severe exacerbation.

### Covariates

Baseline characteristics were assessed on the index date and included age, sex, socioeconomic status (SES), smoking history, exacerbation history,^24^ comorbidities, medication history, age-adjusted Charlson Comorbidity Index (excluding chronic pulmonary disease in the calculation), and frailty (based on the John Hopkins claims-based frailty indicator^26^). The SES was based on medical coverage, and low SES was defined by increased medical reimbursements, which is provided to individuals with low income. Comorbidities and medication history were identified in the year before the index date using International Classification of Diseases (ICD)-coded diagnoses, medical procedure codes, medication use, or a combination thereof. More information about the covariates can be found in e-Table 2.

### Statistical Analyses

Descriptive statistics at index date included the median with interquartile range for continuous variables and frequencies with percentages for categorical variables. Crude event rates with 95% CIs were calculated as the number of (first) exacerbations per 100 person-years at risk, reported for all exacerbations (moderate and severe) and separately for severe exacerbations. Cumulative incidence was estimated using the Aalen-Johansen estimator, accounting for death as competing risk.

Cox proportional hazard analyses were used to assess the association between PPI use and no PPI use and risk of exacerbations. Besides an unadjusted model, model 1 was adjusted for age and sex; model 2 additionally included smoking history and SES; model 3 added exacerbation history and short-acting bronchodilator use; and model 4 further adjusted for frailty, Charlson Comorbidity Index, and GERD diagnosis (defined using ICD codes and medical procedure codes). To minimize confounding by indication, we repeated analyses using stabilized inverse probability of treatment weighting (IPTW) for overall PPI use as well as for each PPI dose category separately given that different doses could have different indications. Propensity scores were calculated with logistic regression models, including the 23 confounding covariates described in Table 1. Stabilized weights were calculated using average effect of treatment based on propensity score and were trimmed at 0.5th and 99.5th percentile. Covariate balance before and after weighting was checked using standardized mean differences, with differences of < 0.1 indicating a good balance, graphically represented in love plots (e-Fig 2).^27^ Weighted Cox regression analyses were used to estimate adjusted hazard ratios (HRs) with 95% CIs. To address residual confounding, covariates that remained imbalanced after IPTW were included in the model.^28^ Additionally, all analyses were repeated in patients with a hospital discharge diagnosis of asthma (ICD, 10th Revision, code J45), COPD (ICD, 10th Revision, code J44), and asthma-COPD overlap (ACO; ICD, 10th Revision, codes J44 and J45). The proportional hazards assumption was checked visually using scaled Schoenfeld residuals. No multicollinearity between factors was observed. A 2-sided *P* value of < .05 was considered statistically significant. All analyses were performed in R Studio version 4.3.0 (R Foundation for Statistical Computing).

**Table 1:** Baseline Characteristics of the Study Population

Characteristic	Overall (N = 932,135)	No PPI Use (n= 516,048)	PPI Use (n = 416,087)
Female sex	501,973 (53.9)	262,694 (50.9)	239,279 (57.5)
Age, y	61.3 (46.3-73.9)	56.5 (40.0-70.1)	66.2 (54.0-77.8)
Low SES	269,946 (29.0)	122,834 (23.8)	147,112 (35.4)
Ever smoking status	216,348 (23.2)	88,637 (17.2)	127,711 (30.7)
Weight			
Normal	867,198 (93.0)	498,407 (96.6)	368,791 (88.6)
Cachexia or underweight	9,359 (1.0)	2,248 (0.4)	7,111 (1.7)
Obesity or overweight	55,578 (6.0)	15,393 (3.0)	40,185 (9.7)
Exacerbation history			
None	326,934 (35.1)	214,800 (41.6)	112,134 (26.9)
1 moderate	259,253 (27.8)	146,699 (28.4)	112,554 (27.1)
≥ 2 moderate or 1 severe	345,948 (37.1)	154,549 (29.9)	191,399 (46.0)
Comorbidities			
Arthritis	22,024 (2.4)	4,761 (0.9)	17,263 (4.1)
Bronchiectasis	3,436 (0.4)	875 (0.2)	2,561 (0.6)
Cancer	54,663 (5.9)	16,933 (3.3)	37,730 (9.1)
Cardiovascular comorbidity	328,899 (35.3)	128,291 (24.9)	200,608 (48.2)
Depression or anxiety	235,435 (25.3)	91,194 (17.7)	144,241 (34.7)
Diabetes mellitus	149,988 (16.1)	58,415 (11.3)	91,573 (22.0)
Frailty	121,468 (13.0)	41,934 (8.1)	79,534 (19.1)
GERD	23,987 (2.6)	1,278 (0.2)	22,709 (5.5)
Sleep apnea	39,012 (4.2)	15,342 (3.0)	23,670 (5.7)
Stomach ulcer	12,071 (1.3)	888 (0.2)	11,183 (2.7)
Medication use			
Acetylsalicylic acid	209,454 (22.5)	82,258 (15.9)	127,196 (30.6)
Antacid	50,061 (5.4)	16,224 (3.1)	33,837 (8.1)
Clopidogrel	32,124 (3.4)	10,125 (2.0)	21,999 (5.3)
DOAC	61,243 (6.6)	21,479 (4.2)	39,764 (9.6)
NSAID	389,458 (41.8)	192,181 (37.2)	197,277 (47.4)
SABD use^a^			
Appropriate	804,307 (86.3)	446,583 (86.5)	357,724 (86.0)
Overuse	70,832 (7.6)	39,806 (7.7)	31,026 (7.5)
Heavy overuse	56,996 (6.1)	29,659 (5.7)	27,337 (6.6)
Vitamin K antagonist	24,866 (2.7)	9,914 (1.9)	14,952 (3.6)

Data are presented as No. (%) or median (interquartile range). DOAC = direct oral anticoagulant; GERD = gastroesophageal reflux disease; NSAID = nonsteroidal antiinflammatory drug; PPI = proton pump inhibitor; SABD = short-acting bronchodilator; SES = socioeconomic status.

a Appropriate use, 0-2 canisters/y; overuse, 3-5 canisters/y; heavy overuse, ≥ 6 canisters/y.

### Supplementary Analyses

To assess the robustness of the results, we first performed stratified IPTW analyses in patients with and without a GERD diagnosis, across age groups (18-50 years, ≥ 50-80 years, and ≥ 80 years) and in patients with and without frailty. Second, to be more stringent regarding exposure, we limited PPI users to those whose accumulated DDDs covered the index date. Additionally, for users already filling their last PPI prescription at baseline, follow-up was censored at the calculated DDD end date, and for initial patients who did not use PPIs, follow-up was also censored if they started using a PPI (at the date of the first prescription of a PPI during follow-up). Third, specifically to assess the effect of PPI use of ≤ 1 week, we subdivided the 1-28 DDD category into 1-7 DDDs and 8-28 DDDs. Fourth, within the stringent PPI user population on index date, we performed a Cox regression analysis to examine the effect of concurrent CYP2C19 inhibitor use on index date, because these drugs increase PPI concentrations (e-Table 2). Fifth, we restricted the Cox analyses to severe (hospitalization-defined) exacerbations to assess whether the observed associations persisted.

## Results

### Baseline Characteristics

A total of 932,135 patients (with a total of 1,084,446 years of follow-up) were included in this study (e-Fig 3). Among the total population, 416,087 patients (44.6%) were receiving PPIs (Table 1). Compared with those not receiving PPIs, patients in the PPI group were more likely to be female (57.5% vs 50.9%), to be older (median, 66.2 years [interquartile range, 54.0-77.8 years] vs 56.5 years [interquartile range, 40.0-70.1 years]), to have lower SES (35.4% vs 23.8%), to have ever smoked (30.7% vs 17.2%), to have more exacerbations at baseline (46.0% vs 29.9% experienced ≥ 2 moderate or 1 severe exacerbation in the previous year), and to have more comorbidities such as cardiovascular comorbidities (48.2% vs 24.9%). Additionally, patients were classified by cumulative PPI exposure during the baseline year. In total, 516,048 patients (55.4%) had no PPI use, 57,540 patients (6.2%) had received ≤ 28 DDDs, 128,017 patients (13.7%) had received 29-180 DDDs, 127,981 patients (13.7%) had received 181-365 DDDs, and 102,549 patients (11.0%) had received > 365 DDDs. Baseline characteristics of patients for each PPI dose category are shown in e-Table 3.

### Association Between PPI Use and Exacerbations

**Figure 1:**
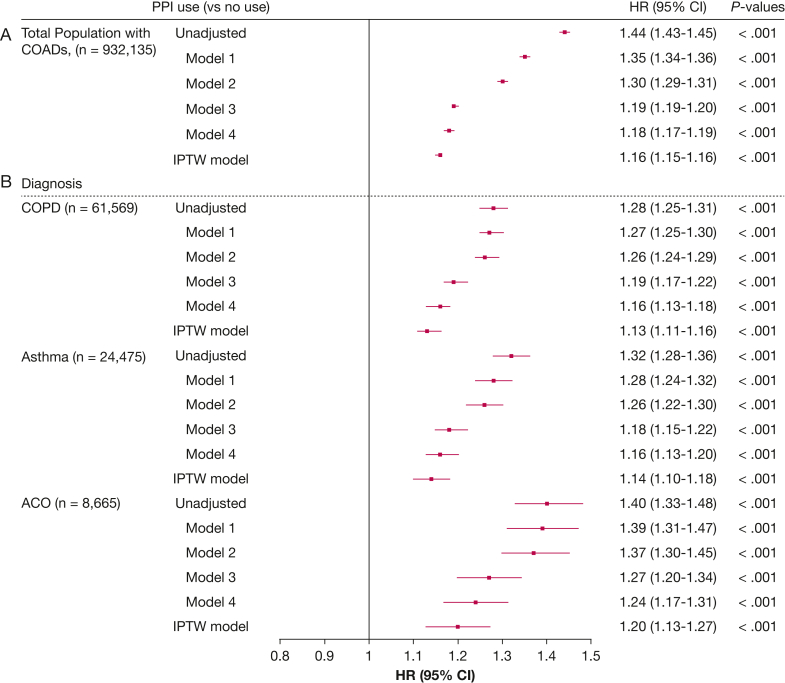
A, B, Forest plots showing Cox regression analyses of the association between PPI use and exacerbations: associations for the total population with COAD (A) and associations stratified by COPD, asthma, and ACO diagnosis (B). Model 1: adjusted for age and sex. Model 2: model 1 plus smoking history and socioeconomic status. Model 3: model 2 plus exacerbation history and short-acting bronchodilator use. Model 4: model 3 plus frailty status, age-adjusted Charlson Comorbidity Index and gastroesophageal reflux disease. IPTW model: propensity scores were calculated with logistic regression models including 23 confounding covariates. ACO = asthma-COPD overlap; COAD = chronic obstructive airway disease; HR = hazard ratio; IPTW = inverse probability of treatment weighting; PPI = proton pump inhibitor.

**Figure 2:**
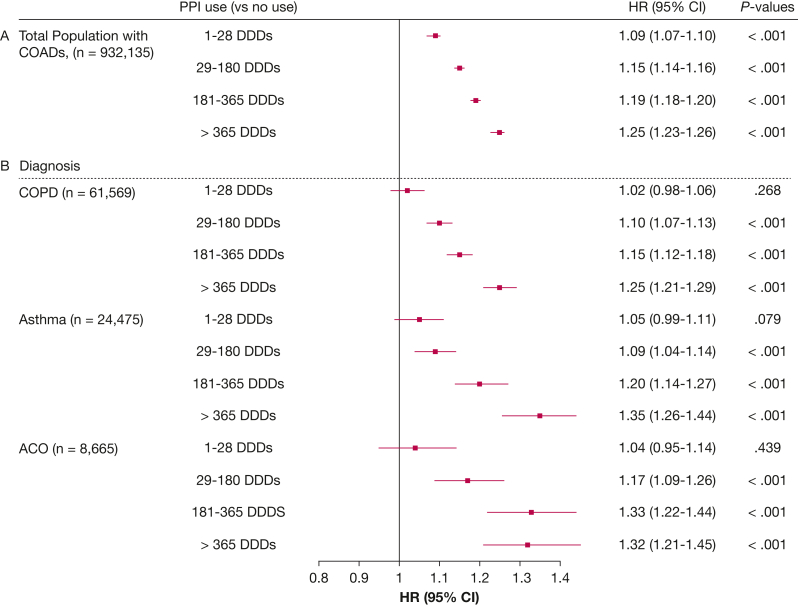
A, B, Forest plots showing weighted Cox regression analyses of the dose-dependent association between proton pump inhibitor use and exacerbations: associations for the total population with COAD (A) and associations stratified by COPD, asthma, and ACO diagnosis (B). Propensity scores were calculated with logistic regression models including 23 confounding covariates. ACO = asthma-COPD overlap; COAD = chronic obstructive airway disease; DDD = defined daily dose; HR = hazard ratio; PPI = proton pump inhibitor.

The crude exacerbation rate (moderate and severe) was 49.3 per 100 person-years at risk among patients who did not use PPIs, compared with 77.7 per 100 person-years at risk among PPI users (Table 2). The crude rate of severe exacerbations is presented in e-Table 4. The cumulative incidence curves of all exacerbations and of severe exacerbations by PPI (dose) are illustrated in e-Figures 4 and 5. In a Cox proportional hazards analysis adjusted for age, sex, smoking history, SES, exacerbation history, short-acting bronchodilator use, frailty, Charlson Comorbidity Index, and presence of GERD, PPI use was associated with an increased exacerbation risk (model 4: adjusted HR, 1.18 [95% CI, 1.17-1.19]) (Fig 1A). To minimize confounding by indication, IPTW-weighted Cox regression was applied, which confirmed this association (HR, 1.16 [95% CI, 1.15-1.16]) (Fig 1A). When stratifying by diagnosis, more similar estimates were found for COPD and asthma (COPD IPTW model: HR, 1.13 [95% CI, 1.11-1.16]; asthma IPTW model: HR, 1.14 [95% CI, 1.10-1.18]) (Fig 1B), whereas in patients with ACO, estimates were slightly higher (ACO IPTW model: HR, 1.20 [95% CI, 1.13-1.27]) (Fig 1B). To assess dose-dependent associations between PPI use and exacerbations, additional weighted Cox regression analyses were performed (Fig 2A). These IPTW-adjusted models showed a progressively increasing exacerbation risk across higher PPI use categories. Compared with no PPI use, the HRs were 1.09 (95% CI, 1.07-1.10), 1.15 (95% CI, 1.14-1.16), 1.19 (95% CI, 1.18-1.20), and 1.25 (95% CI, 1.23-1.26) for up to 28, 180, 365, and > 365 DDDs of PPI, respectively. Stratification by COPD, asthma, or ACO showed sligthly stronger estimates for long-term PPI use in asthma and ACO compared with COPD (Fig 2B).

**Table 2:** Number of Patients and Crude Event Rates With 95% CIs per 100 Person-Years at Risk for Each PPI Dose Category

Variable	No. of Patients (%)	Exacerbation Rate per 100 Person-Years At Risk (95% CI)
No PPI use	516,048 (55.4)	49.3 (49.1-49.5)
Overall PPI use, DDD	416,087 (44.6)	77.7 (77.4-77.9)
1-28	57,540 (6.2)	64.0 (63.4-64.6)
29-180	128,017 (13.7)	72.7 (72.2-73.2)
181-365	127,981 (13.7)	81.1 (80.6-81.6)
> 365	102,549 (11.0)	89.9 (89.3-90.6)

DDD = defined daily dose; PPI = proton pump inhibitor.

### Supplementary Analyses

First, when stratifying the weighted Cox regression analyses by GERD diagnosis, the overall association was significant in both strata (GERD: HR, 1.15 [95% CI, 1.08-1.23] vs no GERD: HR, 1.16 [95% CI, 1.15-1.16]). The dose-dependent pattern persisted across the strata. However, 1-28 DDDs and 29-180 DDDs of PPI were not statistically associated with exacerbations among patients with GERD (1-28 DDDs: HR, 0.97 [95% CI, 0.87-1.08]; 29-180 DDDs: HR, 1.07 [95% CI, 1.00-1.15]), whereas it was significant in patients without GERD (1-28 DDDs: HR, 1.09 [95% CI, 1.07-1.10]; 29-180 DDDs: HR, 1.15 [95% CI, 1.14-1.16]), and the dose-dependent association was more pronounced in patients without GERD (e-Fig 6). When we stratified by age group, the strongest effect of PPIs on exacerbations was observed in the youngest age group (e-Fig 7). Stratification by frailty demonstrated stronger associations in patients without frailty, although associations in both strata remained significant (e-Fig 8). Second, when being more stringent on actual PPI exposure at index date, the adjusted HR was 1.26 (95% CI, 1.26-1.27) (e-Fig 9, model 4) compared with the estimates of the overall analysis (model 4: adjusted HR, 1.18 [95% CI, 1.17-1.19]). Third, when evaluating PPI exposure of ≤ 1 week, the association was not statistically significant (HR, 1.02 [95% CI, 0.99-1.05]) (e-Fig 10). Fourth, 7,952 active PPI users (2.9%) on index date were using a CYP2C19 inhibitor concurrently. PPI users who were taking a CYP2C19 inhibitor simultaneously (mainly fluoxetine or fluconazole) showed an additional 17% increased exacerbation risk (HR, 1.17 [95% CI, 1.14-1.20]) (e-Fig 11, model 4) compared with PPI users not taking a CYP2C19 inhibitor simultaneously. Fifth, when restricting the analyses to severe exacerbations, the associations remained statistically significant (HR, 1.09 [95% CI, 1.07-1.12]) (e-Fig 12, IPTW model).

## Discussion

In this nationwide cohort study conducted between 2017 and 2022, we included 932,135 patients with COADs, of whom 44.6% used PPIs. We found an increased exacerbation risk in patients using PPIs, particularly among long-term users. Associations were most pronounced in younger patients, in those without GERD, in patients without frailty, and in long-term PPI users with asthma (overlap).

Our findings showed that PPI use is associated with an increased risk of exacerbations, even after minimizing confounding by indication using IPTW. This aligns with a European observational cohort study reporting that PPI use was significantly associated with a shorter time to severe exacerbation in patients with COPD.^14^ Similarly, an observational cohort study conducted across 12 countries found that PPI use was associated with an increased risk of COPD exacerbations, also in the absence of a GERD diagnosis.^16^ In patients with asthma, a systematic review and meta-analysis showed that PPI use may contribute to both the development and exacerbation of asthma.^29^ Furthermore, a randomized controlled trial found that children with a phenotype of poor CYP2C19 metabolization who were taking PPIs showed worse asthma control. This phenotype results in reduced metabolism of PPIs and higher drug concentrations.^30^ These findings align with our observation of a higher exacerbation risk in PPI users with concomitant CYP2C19 inhibitor use compared with those not using a CYP2C19 inhibitor. Additionally, we observed that the effect of PPI use on exacerbations decreased with age, which aligns with findings from an observational cohort study on the effect of PPI use on pneumonia in patients with COPD, potentially explained by age-related competing risks.^31^ In line, a sensitivity analysis more stringent on PPI exposure showed even stronger effect estimates.

Several hypotheses have been proposed to explain the increased exacerbations risk in PPI users. First, PPIs may cause overgrowth of pathogenic bacteria, leading to gut dysbiosis.^32^ This microbial imbalance is associated with increased systemic and airway inflammation and may influence lung immunity and health.^33^ Additionally, gut dysbiosis may influence respiratory microbiota through translocation of gut microbiota to the lungs and through altered circulating inflammatory cytokines leading to lung dysbiosis, which may affect lung function negatively.^34^ This mechanism highlights the importance of the role of the gut-lung axis in development and progression of COADs.^34^ Second, direct damage has been proposed because proton pumps are presented in (endothelial) lysosomes.^35^ PPIs may impair lysosomal acidification, enzyme activity, and proteostasis, leading to the accumulation of protein aggregates.^36^ This is associated with increased oxidative stress, endothelial dysfunction, and accelerated endothelial aging, which may increase vascular permeability and thereby contribute to poor COAD control.^36^, ^37^, ^38^ Third, a low gastric pH is essential for converting pepsinogen into active pepsin, which breaks down proteins. PPIs raise gastric pH, hindering this activation.^39^ As a result, incompletely digested proteins may act as antigens and may induce T-helper 2-response and IgE sensitization of the immune system, potentially triggering asthma exacerbations in susceptible people.^39^, ^40^, ^41^ Fourth, PPIs are associated with an increased risk of vitamin and mineral deficiencies, which may contribute further to an increased exacerbation risk.^42^

GERD significantly increases exacerbation risk, and addressing this indication by PPI is expected to reduce exacerbation risk, as observed previously.^15^^,^^43^ However in a randomized controlled trial by Sasaki et al^15^ excluding patients with GERD, lansoprazole still reduced the number of exacerbations compared with control participants, although no placebo was used, sample size was small, and the PPI group also included borderline significantly more people vaccinated against influenza. PPIs are indicated to treat conditions related to excessive gastric acid such as the rare Zollinger-Ellison syndrome or more commonly GERD. GERD is a prevalent comorbidity in patients with COADs and is a risk factor for exacerbations.^7^^,^^12^^,^^13^ Two mechanisms may underlie this association: (1) microaspiration of gastric contents, causing damage to the airways, and (2) vagal nerve stimulation triggered by reflux leading to bronchoconstriction.^7^ Additionally, patients with COAD may have increased intra-abdominal pressure and negative intrathoracic pressure, may cough more frequently, and may use β_2_-agonists, which further decrease lower esophageal sphincter pressure.^7^^,^^12^ In our study, we found that short-term PPI use in patients with a GERD diagnosis was not significantly associated with an increased exacerbation risk, indicating that low-dose short-term use in well-established indications may be appropriate.^44^ Additionally, PPI use of ≤ 1 week was not associated with increased exacerbation risk in the total population with COADs. These findings support that short-term use may be clinically justified. This contrasts with findings from a Taiwanese observational study in which high-dose PPI use was associated with a reduced risk of acute exacerbations in COPD with symptomatic GERD compared with low-dose use.^45^

Among the population of adults with respiratory impairment (median age, 61 years), 44.6% used PPIs in the year before inclusion, compared with 20.6% in the total Belgian population based on 2023 public pharmacy data.^46^ Although the average older age of the study population (61 years vs 41 years) may contribute to this markedly higher PPI use,^47^^,^^48^ PPIs are frequently used in patients with asthma, COPD, or both^7^ and are often overused, leading to unnecessary costs, avoidable adverse events, and drug-drug interactions.^10^ Indeed, our study demonstrated for the first time to our knowledge that the exacerbation risk associated with PPI use is elevated when combined with a CYP2C19 inhibitor, theoretically increasing PPI plasma concentrations. Given the widespread and often chronic use of PPIs, a better understanding of the risk-benefit balance is crucial to incorporate in prescribing practices. Future research should aim to clarify the mechanisms underlying the association between PPI use and COAD exacerbations, particularly the role of gut-lung axis in patients with COADs. Additionally, large randomized controlled trials are needed to state causality of any beneficial effects of PPI use in real-life patients with COAD and GERD.

The strengths of this nationwide cohort study include the large sample size of patients with respiratory impairment among the general population because of the compulsory health insurance, reducing selection and recall bias, and the long-term prospective follow-up period. Additionally, both ambulatory and hospital care data were used to define PPI exposure and additionally to identify comorbidities. To reduce confounding by indication, we conducted stabilized IPTW analyses, adjusting for a wide range of confounders. Furthermore, we found higher exacerbation risk among long-term PPI users and those with theoretically higher PPI concentrations, which strengthens the validity of the association. Nevertheless, some limitations should be acknowledged. First, PPI use was based on dispensing data, not on the actual intake, which may lead to overestimation. In contrast, PPI use was potentially underestimated because of small packages of low dose PPIs being available over the counter in Belgium without reimbursement, and dispensing data of these deliveries were not captured in the nationwide database. Because of the observational study design, coding errors and misclassification bias are possible. Factors such as obesity or being overweight, cachexia or being underweight, and smoking also may be underreported. However, we addressed this limitation by using broad definitions that incorporated ICD codes, medical procedure codes, and medication use. Second, information on disease severity was lacking. We mitigated this limitation by incorporating proxies for disease severity, such as exacerbation history and short-acting bronchodilator use, which indeed seemed to modify the association partly. Third, despite adjustment for 23 confounders using stabilized IPTW analyses, residual confounding by unmeasured factors cannot be excluded fully. For example, if smoking was not registered, it may still have influenced results, because smoking is linked to increased gastric acid production and reflux.^49^ Fourth, our findings are limited to patients treated for obstructive lung diseases and cannot be generalized to those not receiving respiratory medication.

## Interpretation

PPI use in patients with COADs was associated with an increased exacerbation risk, especially among long-term users. This highlights the need to carefully (re)consider their use in clinical practice, particularly in patients without an ongoing clear indication.

## Funding/Support

The authors have reported to *CHEST* that no funding was received for this study.

## Financial/Nonfinancial Disclosures

The authors have reported to *CHEST* the following: L. L. has been a consultant for AstraZeneca, GlaxoSmithKline, and Sanofi and has given lectures sponsored by Chiesi, IPSA vzw, and Domus Medica vzw (nonprofit organizations facilitating lifelong learning for health care providers), all paid to her institution; has received support for travel from Menarini and AstraZeneca; is an unpaid member of the European Respiratory Society and Belgian Respiratory Society; and is a member of the Faculty Board of Ghent University Faculty of Pharmaceutical Sciences and faculty committees. V. D. and F. V. V. are unpaid members of European Respiratory Society and Belgian Respiratory Society. None of these are related to the content of this work. None declared (K. V.).

## Supplementary Data

e-Online Data

Audio
