# Supplementary material for: Exacerbation Risk by Chronic Proton Pump Inhibitor Use in Obstructive Lung Diseases
Source: Chest. 2026 Jan 16;169(5):1218–27. doi: 10.1016/j.chest.2026.01.002 (PMC13197971; doi:10.1016/j.chest.2026.01.002)
Supplement: e-Online Data [file mmc1.docx]

**Supplementary Materials**

Table of Contents

[Supplemental methods 3](#_Toc224631566)

[Supplemental tables 4](#_Toc224631567)

[e-Table 1: STROBE reporting guideline 4](#_Toc224631568)

[e-Table 2: Definition of in- and exclusion criteria, exposure, demographics, comorbidities, comedication, clinical risk scores and outcomes 6](#_Toc224631569)

[e-Table 3: Baseline characteristics of the study population stratified by PPI dose category 14](#_Toc224631570)

[e-Table 4: The number of patients and crude event rates for severe exacerbations with 95% CI per 100 person-years at risk for each PPI dose category 16](#_Toc224631571)

[Supplemental figures 17](#_Toc224631572)

[e-Figure 1: Overview of the study design 17](#_Toc224631573)

[e-Figure 2: Love plots 18](#_Toc224631574)

[e-Figure 3: Flowchart of study population. 23](#_Toc224631575)

[e-Figure 4: Cumulative incidence of exacerbations in COAD patients by PPI (dose) 24](#_Toc224631576)

[e-Figure 5: Cumulative incidence of severe exacerbations in COAD patients by PPI (dose) 26](#_Toc224631577)

[e-Figure 6: Weighted Cox regression analyses of the (dose-dependent) association between proton pump inhibitor use and exacerbations stratified by GERD diagnosis 28](#_Toc224631578)

[e-Figure 7: Weighted Cox regression analyses of the dose-dependent association between proton pump inhibitor use and exacerbations stratified by age groups 29](#_Toc224631579)

[e-Figure 8: Weighted Cox regression analyses of the dose-dependent association between proton pump inhibitor use and exacerbations stratified by frailty status 30](#_Toc224631580)

[e-Figure 9: Cox regression analyses of the association between actual proton pump inhibitor use on index date and exacerbations, with censored follow-up (n = 786,721) 31](#_Toc224631581)

[e-Figure 10: Weighted Cox regression analyses of the dose-dependent association between proton pump inhibitor use and exacerbations, with short-term PPI use divided (≤ 1 week vs. > 1 week) (n = 932,135) 32](#_Toc224631582)

[e-Figure 11: In PPI users on index date, Cox regression analyses of the association between simultaneous CYP2C19 inhibitor use and exacerbations, with censored follow-up (n = 244,318) 33](#_Toc224631583)

[e-Figure 12: Cox regression analyses of the association between proton pump inhibitor use and severe exacerbations (n = 932,135) 34](#_Toc224631584)

[References 35](#_Toc224631585)

# Supplemental methods

The source population was provided by the InterMutualistic Agency (IMA) and Minimal Hospital Dataset (MHD). The IMA centralizes all claims data from Belgian health insurance funds and manages three databases: (1) a population database containing socio-demographic characteristics (e.g. age and sex), (2) a healthcare database containing data on reimbursed ambulatory and hospital care (e.g. medical procedures, inpatient medication and other reimbursed care) and (3) a pharmaceutical database containing outpatient medication prescription claims (e.g., dispensing date, Anatomical Therapeutic Chemical [ATC] Classification code). Data are available for all legal residents who are affiliated with a Belgian health insurance fund^1,2^. MHD, collected by the Belgian Ministry of Health, collects discharge diagnoses for each hospital admission, coded in International Classification of Diseases (ICD) codes (ICD-10)^3^. Data from IMA and MHD were linked by a Trusted Third Party (eHealth)^1^.

# Supplemental tables

## e-Table 1: STROBE reporting guideline

|  | **Item No.** | **Recommendation** | **Page No.** |
| --- | --- | --- | --- |
| **Title and abstract** | 1 | (*a*) Indicate the study’s design with a commonly used term in the title or the abstract | 3 |
|  |  | (*b*) Provide in the abstract an informative and balanced summary of what was done and what was found | 3 |
| **Introduction** | | |  |
| Background/rationale | 2 | Explain the scientific background and rationale for the investigation being reported | 4 |
| Objectives | 3 | State specific objectives, including any prespecified hypotheses | 4 |
| **Methods** | | |  |
| Study design | 4 | Present key elements of study design early in the paper | 4, Supplemental methods |
| Setting | 5 | Describe the setting, locations, and relevant dates, including periods of recruitment, exposure, follow-up, and data collection | 5 |
| Participants | 6 | (*a*) Give the eligibility criteria, and the sources and methods of selection of participants. Describe methods of follow-up | 5, e-Figure 1 & 2 |
|  |  | (*b*) For matched studies, give matching criteria and number of exposed and unexposed | / |
| Variables | 7 | Clearly define all outcomes, exposures, predictors, potential confounders, and effect modifiers. Give diagnostic criteria, if applicable | 5-7 |
| Data sources/ measurement | 8* | For each variable of interest, give sources of data and details of methods of assessment (measurement). Describe comparability of assessment methods if there is more than one group | 5, e-Table2 |
| Bias | 9 | Describe any efforts to address potential sources of bias | 6-8 |
| Study size | 10 | Explain how the study size was arrived at | 5, e-Figure 3 |
| Quantitative variables | 11 | Explain how quantitative variables were handled in the analyses. If applicable, describe which groupings were chosen and why | 5-7 |
| Statistical methods | 12 | (*a*) Describe all statistical methods, including those used to control for confounding | 7-8 |
|  |  | (*b*) Describe any methods used to examine subgroups and interactions | 8 |
|  |  | (*c*) Explain how missing data were addressed | / |
|  |  | (*d*) If applicable, explain how loss to follow-up was addressed | / |
|  |  | (*e*) Describe any sensitivity analyses | 8 |
| **Results** | | |  |
| Participants | 13* | (a) Report numbers of individuals at each stage of study—eg numbers potentially eligible, examined for eligibility, confirmed eligible, included in the study, completing follow-up, and analysed | 9-11, e-Figure 3 |
|  |  | (b) Give reasons for non-participation at each stage | / |
|  |  | (c) Consider use of a flow diagram | e-Figure 3 |
| Descriptive data | 14* | (a) Give characteristics of study participants (eg demographic, clinical, social) and information on exposures and potential confounders | Table 1, e-Table 3 |
|  |  | (b) Indicate number of participants with missing data for each variable of interest | / |
|  |  | (c) Summarise follow-up time (eg, average and total amount) | 8 |
| Outcome data | 15* | Report numbers of outcome events or summary measures over time | Table 2, e-Figure 4 |
| Main results | 16 | (*a*) Give unadjusted estimates and, if applicable, confounder-adjusted estimates and their precision (eg, 95% confidence interval). Make clear which confounders were adjusted for and why they were included | 11, Figure 1 & 2,  e-Figure 5-9 |
|  |  | (*b*) Report category boundaries when continuous variables were categorized | Tabel 2 |
|  |  | (*c*) If relevant, consider translating estimates of relative risk into absolute risk for a meaningful time period | / |
| Other analyses | 17 | Report other analyses done—eg analyses of subgroups and interactions, and sensitivity analyses | 12-13, e-Figure 5 - 9 |
| **Discussion** | | |  |
| Key results | 18 | Summarise key results with reference to study objectives | 13 |
| Limitations | 19 | Discuss limitations of the study, taking into account sources of potential bias or imprecision. Discuss both direction and magnitude of any potential bias | 16-17 |
| Interpretation | 20 | Give a cautious overall interpretation of results considering objectives, limitations, multiplicity of analyses, results from similar studies, and other relevant evidence | 13-17 |
| Generalisability | 21 | Discuss the generalisability (external validity) of the study results | 17 |
| **Other information** | | |  |
| Funding | 22 | Give the source of funding and the role of the funders for the present study and, if applicable, for the original study on which the present article is based | 22 |

## e-Table 2: Definition of in- and exclusion criteria, exposure, demographics, comorbidities, comedication, clinical risk scores and outcomes

| **VARIABLE** | **ICD, ATC AND MEDICAL PROCEDURE CODES** |
| --- | --- |
| **INCLUSION CRITERIA^4,5^** | |
| Drugs for obstructive airway diseases | **ATC:** R03 |
| ≥18 years | Age ≥18 years on index date |
| ≥1 year coverage | ≥1 year coverage by a Belgian health insurance fund |
| **EXCLUSION CRITERIA** | |
| Liver cirrhosis | **ICD-10:** K74.3 – K74.6, K70.3, K71.7 |
| Methotrexate | **ATC:** L01BA01, L04AX03 |
| Histamine type-2 receptor antagonists | **ATC:** A02BA |
| **EXPOSURE** | |
| Proton pump inhibitor | **ATC:** A02BC |
| **DEMOGRAFICS (on index date)^4-6^** | |
| Age | Age on index date based on the year and month of birth, not the exact date due to patient privacy. |
| Sex | Sex on index date |
| (Ever-)smoker | Patients were categorized as (ever-)smoking if any ICD code (e.g. (history of) nicotine dependence) (ICD-9 until 2014, ICD-10 since 2015), ATC code (N07BA, N06AX12: drugs used in nicotine dependence) or medical procedure code (e.g. tobacco counselling) was registered between January 1st, 2010 and the index date.  **ICD-9:** 305.1, V15.82  **ICD-10:** F17, Z71.6, Z72.0, Z87.891  **ATC**: N07BA, N06AX12  **Medical procedure code:** 740434, 740445, 740456, 740460, 740471, 740482 |
| **CHARACTERISTICS (≤1 year before index date)** | |
| Exacerbation history | **Moderate exacerbations**:  Outpatient prescription fill for an oral corticosteroid (ATC: H02AB, except parental use (defined on package level)) and/or guideline-recommended antibiotics (ATC: 01CA, J01CR, J01DC, J01FA, J01MA, except continuous azithromycin therapy). Prescription fills needed to be separated by at least 14 days to be considered as separate events. Moderate exacerbations that led to hospital admission for a severe exacerbation within 14 days were classified only as hospitalized exacerbations, to avoid counting the same event twice.  **Severe/hospitalized exacerbations**:  Hospital admissions or emergency department visits with a primary diagnosis code of asthma or (acute) COPD exacerbation, COPD with acute lower respiratory infection or asthma with status asthmaticus (ICD-10: J44.0, J44.1, J45.21, J45.22, J45.31, J45.32, J45.41, J45.42, J45.51, J45.52, J45.901 or J45.902) OR with a primary diagnosis of chronic lower respiratory disease (ICD-10: J40-J47) or respiratory failure (ICD-10: J96), combined with a secondary diagnosis code of asthma or (acute) COPD exacerbation, COPD with acute lower respiratory infection or asthma with status asthmaticus (ICD-10: J44.0, J44.1, J45.21, J45.22, J45.31, J45.32, J45.41, J45.42, J45.51, J45.52, J45.901 or J45.902). Hospitalizations with a concomitant pneumonia diagnosis were excluded (ICD-10: A01.03, A02.22, A37.01, A37.11, A37.81, A37.91, A50.04, A54.84, B01.2, B05.2, B06.81, B77.81, J09.X1, J09.X2, J09.X3, J10.0, J11.0, J12, J13, J14, J15, J16, J17, J18, J84.11, J84.2, J85.1, J95.851).  Patients were categorized into subgroups based on their exacerbation history in the year before the index date:  1) no exacerbations  2) one moderate exacerbation  3) two or more moderate exacerbation and/or one severe exacerbation. |
| **COMORBIDITIES (≤1 year before index date)**^4-6^ | |
| **RESPIRATORY** | |
| COPD | **ICD 10:** J44 |
| Asthma | **ICD 10:** J45 |
| Pneumonia | **ICD-10:** A01.03, A02.22, A37.01, A37.11, A37.81, A37.91, A50.04, A54.84, B01.2, B05.2, B06.81, B77.81, J09.X1, J09.X2, J09.X3, J10.0, J11.0, J12, J13, J14, J15, J16, J17, J18, J84.11, J84.2, J85.1, J95.851, Z87.01 |
| Sleep apnea | **ICD-10:** G47.3  **Medical procedure code:** 765951, 779870, 779881, 779892, 779903, 779914, 779925, 779936, 779951, 788012, 788023 |
| Bronchiectasis | **ICD-10:** J47 |
| Cystic fibrosis | **ICD-10:** E84  **ATC**: R05CB13, R07AX02, R07AX30, R07AX31, R07AX32  **Medical procedure code:** 604450, 604472, 604494, 604516, 604531, 604553 |
| **CANCER** | |
| Cancer | **ICD-10:** C00-C96, Z51.0, Z51.11, Z51.12  **ATC:** L01  **Medical procedure code:** 154873, 154884, 154895, 154906, 157231, 157242, 201191, 201202, 201213, 201224, 220275, 220286, 220371, 220382, 226914, 226925, 226936, 226940, 227216, 227220, 227275, 227286, 227636, 227640, 227651, 227662, 227673, 227684, 227695, 227706, 227710, 227721, 227732, 227743, 227754, 227765, 227776, 227780, 227791, 227802, 227813, 227824, 227835, 227846, 230473, 230484, 231033, 231044, 235152, 235163, 241231, 241242, 241415, 241426, 241430, 241441, 241452, 241463, 242012, 242023, 242034, 242045, 242292, 242303, 242314, 242325, 242830, 242841, 242852, 242863, 242874, 242885, 242896, 242900, 243051, 243062, 243073, 243084, 243235, 243246, 243736, 243740, 243751, 243762, 243773, 243784, 244016, 244020, 244031, 244042, 244075, 244086, 244311, 244322, 244856, 244860, 244893, 244904, 244915, 244926, 244930, 244941, 244952, 244963, 244974, 244985, 245512, 245523, 245534, 245545, 246050, 246061, 246072, 246083, 247111, 247122, 247133, 247144, 251753, 251764, 251775, 251786, 254892, 254903, 256115, 256126, 256336, 256340, 256572, 256583, 256771, 256782, 257191, 257202, 258355, 258366, 258370, 258381, 258392, 258403, 258451, 258462, 258554, 258565, 258856, 258860, 258871, 258882, 258893, 258904, 259033, 259044, 259114, 259125, 260190, 260201, 260411, 260422, 260433, 260444, 260551, 260562, 260654, 260665, 260750, 260761, 261111, 261122, 261391, 261402, 261472, 261483, 261671, 261682, 261774, 261785, 261796, 261800, 262334, 262345, 262570, 262581, 277756, 277760, 277771, 277782, 278795, 278806, 278810, 278821, 280136, 280140, 280151, 280162, 281831, 281842, 281956, 281960, 282310, 282321, 282671, 282682, 288455, 288466, 288470, 288481, 310494, 310505, 311312, 311323, 312550, 312561, 312572, 312583, 312594, 312605, 312653, 312664, 312970, 312981, 317111, 317122, 350070, 350092, 350114, 350125, 350136, 350140, 350232, 350254, 350265, 350276, 350280, 350291, 350302, 350372, 350383, 350394, 350405, 350416, 350420, 350674, 350685, 350696, 350700, 431174, 431185, 433016, 433020, 435831, 435842, 436295, 436306, 436376, 436380, 444113, 444124, 444135, 444146, 444150, 444161, 444172, 444183, 444194, 444205, 444216, 444220, 444231, 444242, 444253, 444264, 444275, 444286, 444290, 444301, 444312, 444323, 444334, 444345, 444356, 444360, 444371, 444382, 444393, 444404, 444415, 444426, 444430, 444441, 444452, 444463, 444474, 444485, 444496, 444500, 444511, 444522, 444533, 444544, 444555, 444566, 444570, 444581, 444592, 444603, 444636, 444640, 444651, 444662, 444673, 444684, 473970, 473981, 474795, 474806, 532696, 532700, 532711, 532722, 548575, 548586, 565073, 565084, 565095, 565106, 565110, 565121, 565132, 565143, 565154, 565165, 587834, 587845, 587871, 587882, 587893, 587904, 587915, 587926, 588431, 588442, 588453, 588464, 588475, 588486, 588490, 588501, 588512, 588523, 588534, 588545, 588556, 588560, 588571, 588582, 588593, 588604, 588770, 588781, 588976, 588980, 589691, 589702, 589713, 589724, 589831, 589842, 589875, 589886, 594252, 594263, 594274, 594285, 594296, 594300, 594311, 594322, 594333, 594344, 594355, 594366, 594370, 594381, 594392, 594403, 594414, 594425, 594436, 594440, 594451, 594462, 594495, 594506, 594510, 594521, 594532, 594543, 594554, 594565, 594576, 594580, 594591, 594602, 594613, 594624, 594635, 594646, 594694, 594705, 594716, 594720, 594753, 594764, 594775, 594786, 594790, 594801, 594812, 594823, 594834, 594845, 594856, 594860, 594871, 594882, 594893, 594904, 594915, 594926, 594930, 594941, 597273, 597295, 598581, 745010, 745021, 745032, 745043, 745113, 745124, 745135, 745146, 745150, 745161, 771632, 771643 |
| Any malignancy including leukemia and lymphoma | **ICD-10:** C00-C76, C80.1, C80.2, C81-C96, Z51.0, Z51.11, Z51.12  **ATC:** L01  **Medical procedure code:** see ‘Cancer’ |
| Metastatic cancer | **ICD-10:** C77-C79, C80.0 |
| **CARDIOVASCULAR** | |
| Hypertension | **ICD-10:** I10, I11, I12, I13, I15, I16, I67.4  **ATC: combination of treatment of ≥2 of the following drug classes^7^**:   1. **Cardioselective beta blocker:** C07AB, C07FX03, C07FX04, C07FX05, C07FX06 2. **ACE inhibitor or angiotensin II receptor blocker:** C09A, C09BA, C09C, C09DA, C09DX02, C09DX04, C10BX04, C10BX06, C10BX10, C10BX12, C10BX15, C10BX16, C10BX17, C10BX20, C10BX21 3. **Calcium channel blocker:** C08C, C09XA53, C10BX03, C10BX09 4. **Non-loop diuretic:** C03A, C03BA, C03BB, C03EA, C07BA, C07BG, C07DA, C09XA52 5. **Other antihypertensive drugs (alpha adrenergic blocker, vasodilator):** C02A, C02B, C02C, C02DB, C02DD, C02DG, C02L   **or use of ≥1 combination product**: C07BB, C07CB, C07DB, C07FB, C08G, C09BB, C09BX, C09DB, C09DX01, C09DX03, C09DX05, C09DX06, C09DX07, C09DX08, C09XA54, C10BX07, C10BX11, C10BX13, C10BX14, C10BX18, C10BX19 |
| Ischemic heart diseases | **ICD-10:** I20, I21, I22, I23, I24, I25, Z95.1, Z95.5, Z98.61  **Medical procedure code:** 158992, 159003, 159014, 159025, 159036, 159040, 229574, 229585, 229611, 229622, 229633, 229644, 589013, 589024, 589153, 589164, 589934, 589945, 589956, 589960 |
| Myocardial infarction | **ICD-10:** I21, I22, I25.2 |
| Cerebrovascular disease | **ICD-10:** G45, G46, H34.0, I60, I61, I62, I63, I65, I66, I67, I68, I69, Z86.73  **Medical procedure code:** 182136, 182140, 182151, 182162, 182173, 182184, 477724, 477746, 477761, 477783 |
| Prior thromboembolism |  |
| Stroke | **ICD-10:** I61, I62, I63, I67.89, I69.1, I69.2, I69.3, I69.8, I69.9, I97.82, Z86.73  **Medical procedure code:** 182136, 182140, 182151, 182162, 182173, 182184, 477724, 477746, 477761, 477783 |
| Systemic embolism | **ICD-10:** D73.5, I74, I75, I76, K55.0, N28.0  **Medical procedure code:** 235130, 235141, 237112, 237123, 589175, 589186 |
| Pulmonary heart disease and diseases of pulmonary circulation | **ICD-10:** I26, I27, I28, Z86.711  **ATC:** C02KX, B01AC11, B01AC21, B01AC27  **Medical procedure code:** 211540, 211562 |
| Congestive heart failure | **ICD-10:** I09.81, I11.0, I13.0, I13.2, I25.5, I42.0, I42.6-I42.9, I43, I50, P29.0  **ATC: combination of treatment of all of the following drug classes^7^:**   1. **Beta blocker (selective or alpa and beta blocking):** C07AB, C07AG, C07BB, C07BG, C07CB, C07CG, C07DB, C07FB, C07FX03, C07FX04, C07FX05, C07FX06 2. **ACE inhibitor or angiotensin II receptor blocker:** C09A, C09BA, C09BB, C09C, C09DA, C09DB, C09DX01, C09DX02, C09DX03, C09DX04, C09DX06, C09DX07, C09DX08, C10BX04, C10BX06, C10BX07, C10BX10, C10BX11, C10BX12, C10BX13, C10BX14, C10BX15, C10BX16, C10BX17, C10BX18, C10BX19, C10BX20, C10BX21 3. **Loop diuretic:** C03C, C03EB   **or use of ≥1 combination product (beta blocker + ACE inhibitor or beta blocker + angiotensin II receptor blocker)**: C09BX02, C09BX04, C09BX05, C09BX06, C09BX07, C09DX05 **and loop diuretic** |
| Arrythmia | **ICD-10:** I44, I45, I46, I47, I48, I49, R00.0, R00.1, R00.8, T82.1, Z45.0, Z95.0  **ATC:** C01EB10, C01B, C07AA07, C07BA07, C07FX02,  **Medical procedure code:** 180272, 180283, 180294, 180305, 589514, 589525, 589551, 589562 |
| Valvular heart disease | **ICD-10:** A32.82, A39.51, A52.03, A54.83, B33.21, B37.6, I01.1, I05, I06, I07, I08, I09.1, I33, I34, I35, I36, I37, I38, I39, M32.11, Q22, Q23.0, Q23.1, Q23.2, Q23.3, Q23.8, Q23.9, T82.0, T82.2, T82.6, Z95.2, Z95.3, Z95.4, Z95.8  **Medical procedure code:** 159110, 159121, 159132, 159143, 159154, 159165, 159176, 159180, 159191, 159202, 159213, 159224, 159235, 159246, 159250, 159261, 159272, 159283, 159294, 159305, 170634, 170645, 172491, 172502, 172513, 172524, 172734, 172745, 172756, 172760, 172771, 172782, 172955, 172966, 172970, 172981, 172992, 173003, 229515, 229526, 229596, 229600, 589190, 589201, 680153, 680164, 680175, 680186, 680993, 681004, 704616, 704620, 704631, 704642, 704653, 704664 |
| Peripheral vascular disease | **ICD-10:** I70, I71, I73.1, I73.8, I73.9, I74, I77, I79.0, K55.1, K55.8, K55.9, Z95.82, Z98.62  **Medical procedure code:** 229294, 229305, 229316, 229320, 229331, 229342, 235071, 235082, 235093, 235104, 235115, 235126, 235196, 235200, 235211, 235222, 236014, 236025, 236036, 236040, 236051, 236062, 237016, 237020, 237031, 237042, 237053, 237064, 237075, 237086, 237090, 237101, 237171, 237182, 589050, 589061, 589094, 589105, 589175, 589186, 589595, 589606, 589610, 589621, 589632, 589643, 589654, 589665 |
| Anemia | **ICD-10:** D46.0-D46.2, D46.4, D50-D53, D55-D64 |
| **ENDOCRINE, NUTRITIONAL AND METABOLIC** | |
| Diabetes mellitus  (DM) | **ICD-10:** E08, E09, E10, E11, E13, E14, Z46.81, Z96.41  **ATC**: A10  **Medical procedure code:** 102852, 107015, 107030, 107052, 107074, 109594, 174370, 174381, 174392, 174403, 174414, 174425, 174436, 174440, 174451, 174462, 174473, 174484, 174495, 174506, 174510, 174521, 423135, 423150, 423172, 423194, 423216, 423231, 423334, 423813, 423835, 423850, 433554, 433565, 540772, 540783, 543712, 543723, 653671, 653682, 697093, 697104, 754176, 754191, 754250, 754272, 754736, 757352, 757374, 757396, 757411, 757514, 757536, 757551, 757573, 770033, 770055, 770070, 771573, 771595, 773393, 773496, 784630, 784641, 784652, 784663, 785735, 785750, 785772, 785794, 785816, 785831, 785853, 785875, 785890, 785912, 785934, 785956, 786015, 786030, 786100, 788756, 788771, 788793, 788815, 788830, 788852, 788874, 788896, 788911, 788933, 788955, 789751, 789773, 789795, 789810, 789832, 789854, 789876, 789891, 789913, 789935, 794032, 794054, 794076, 794091, 794253, 794275, 794290, 794312, 794334, 794356, 794371, 794393, 794415, 794430, 794452, 961295, 961306, 961332, 961343 |
| Without vascular complications | **ICD-10:** E08.0, E08.1, E08.6, E08.9, E09.0, E09.1, E09.6, E09.9, E10.1, E10.6, E10.9, E11.0, E11.1, E11.6, E11.9, E13.0, E13.1, E13.6, E13.9 |
| With vascular complications | **ICD-10:** E08.2, E08.3, E08.4, E08.5, E08.8, E09.2, E09.3, E09.4, E09.5, E09.8, E10.2, E10.3, E10.4, E10.5, E10.8, E11.2, E11.3, E11.4, E11.5, E11.8, E13.2, E13.3, E13.4, E13.5, E13.8  **Medical procedure code:** 653671, 653682, 697093, 697104, 770070, 773393, 773496 |
| Obesity and overweight^8^ | **ICD-10:** E66, Z68.25-Z68.29, Z68.3, Z68.4  **ATC:** A08AA62, A08AB01  **Medical procedure code:** 241776, 241780, 241813, 241824, 241835, 241846 |
| Cachexia and underweight | **ICD-10:** R64, Z68.1 |
| **GASTROINTESTINAL** | |
| Gastroesophageal reflux disease (GERD) | **ICD-10**: K20, K21, K22.7  **Medical procedure code**: 172616, 172620, 172631, 172642, 172653, 172664 |
| Peptic ulcer disease | **ICD-10**: B96.81, K22.1, K25, K26, K27, K28, Z87.11  **ATC:** A02BD04, A02BD08, A02BD11  **Medical procedure code:** 550093, 550104, 552370, 552381 |
| **GENITOURINARY** | |
| Chronic kidney disease | **ICD-10:** I12.0, I13.11, I13.2, N03.2, N03.3, N03.4, N03.5, N03.6, N03.7, N05.2, N05.3, N05.4, N05.5, N05.6, N05.7, N18, N19, N25.0, T86.1, Z48.22, Z49, Z91.15, Z94.0, Z99.2  **Medical procedure code:** 107096, 107111, 107133, 107155, 318010, 318021, 318290, 318301, 470293, 470304, 470315, 470326, 470330, 470341, 470352, 470374, 470385, 470400, 470422, 470433, 470444, 470466, 470470, 470481, 470875, 470890, 470901, 470912, 470934, 470945, 471111, 471122, 471133, 471144, 471155, 471166, 471170, 471181, 474714, 474725, 754294, 757433, 757492, 761272, 761283, 761456, 761471, 761493, 761515, 761526, 761530, 761552, 761574, 761596, 761655, 761670, 767594, 767616, 767631, 767664, 767686, 767701, 767723, 767734, 767756, 767782, 767804, 767815, 767826, 767830, 767841, 767955, 767966 |
| Urinary tract infection^9^ | **ICD-10:** A56.01, N10, N12, N13.6, N15.1, N15.9, N16, N28.84, N28.85, N28.86, N30.0, N30.8, N30.9, N34, N39.0  **ATC:** J01XE01, J01XX01 |
| **LIVER** | |
| Mild liver disease | **ICD-10:** B17.0, B17.10, B18, B19.10, B19.20, K70.0, K70.1, K70.2, K70.3, K70.9, K71.3, K71.4, K71.5, K71.6, K71.7, K71.8, K71.9, K73, K74, K75.2, K75.3, K75.4, K75.8, K75.9, K76.0, K76.2, K76.3, K76.4, K76.89, K76.9, Z94.4  **ATC:** J05AF07, J05AF08, J05AF10, J05AP  **Medical procedure code:** 318076, 318080, 318334, 318345, 472113, 472124, 556754, 556765, 556776, 556780, 589352, 589363 |
| Moderate and severe liver disease | **ICD-10:** B15.0, B16.0, B16.2, B17.11, B19.0, B19.11, B19.21, I85, I86.4, K70.4, K71.1, K72.1, K72.9, K76.5, K76.6, K76.7 |
| **MOVEMENT RESTRICTIONS** | |
| Hemiplegia/paraplegia | **ICD-10:** G04.1, G11.4, G80.0, G80.1, G80.2, G81, G82, G83.0, G83.4, G83.9  **Medical procedure code:** 643414, 643425 |
| History of falling | **ICD-10:** R29.6, V00.141, V00.811, V00.831, V81.5, V81.6, V82.5, V82.6, V92.0, V93.3, V94.0, V97.0, W00, W01, W03, W05-W15, W16.0-W16.4, W17, W18, W19, Y21.1, Y21.3, Y30, Z91.81 |
| Impaired mobility | **ICD-10:** Z74.01, Z74.09, Z99.3  **Medical procedure code:** 520015, 520026, 520030, 520041, 520052, 520063, 520074, 520085, 520096, 520100, 520111, 520122, 520133, 520144, 520155, 520166, 520170, 520181, 520192, 520203, 520214, 520225, 520310, 520321, 520332, 520343, 520354, 520365, 520376, 520380, 520391, 520402, 520413, 520424, 520435, 520446, 520450, 520461, 520472, 520483, 520494, 520505, 520516, 520520, 520531, 520542, 520553, 520564, 520575, 520586, 520590, 520601, 520612, 520623, 520634, 520645, 520656, 520660, 520671, 520682, 520693, 520704, 520715, 520726, 520730, 520741, 520752, 520763, 520774, 520785, 520796, 520800, 520811, 520822, 520833, 520844, 520855, 520866, 520870, 520881, 520892, 520903, 520914, 520925, 520936, 520940, 520951, 520962, 520973, 520984, 520995, 521006, 521010, 521021, 521032, 521043, 521054, 521065, 521076, 521080, 521091, 521102, 521113, 521124, 521135, 521146, 521150, 521161, 521172, 521183, 521194, 521205, 521216, 521220, 521231, 521242, 521253, 521264, 521275, 521286, 521290, 521301, 521312, 521323, 521334, 521345, 521356, 521360, 521371, 521382, 521393, 521404, 521415, 521426, 521430, 521441, 521452, 521463, 521474, 521485, 521496, 521500, 521511, 521522, 521533, 521544, 521555, 521566, 521570, 521581, 521592, 521603, 521614, 521625, 521636, 521640, 521651, 521662, 521673, 521684, 521695, 521706, 521710, 521721, 521732, 521743, 521754, 521765, 521776, 521780, 521791, 521802, 521813, 521824, 521835, 521846, 521850, 521861, 521872, 521883, 521894, 521905, 521916, 521920, 521931, 521942, 521953, 521964, 521975, 521986, 521990, 522001, 522012, 522023, 522034, 522045, 522056, 522060, 522071, 522082, 522093, 522104, 522115, 522126, 522130, 522141, 522152, 522163, 522174, 522185, 522196, 522200, 522211, 522222, 522233, 522244, 522255, 522266, 522270, 522281, 522292, 522303, 522314, 522325, 522336, 522340, 522351, 522362, 522373, 522384, 522395, 522406, 522410, 522421, 522432, 522443, 522454, 522465, 522476, 522480, 522535, 522550, 522572, 522583, 522594, 522605, 522616, 522620, 522631, 522642, 522653, 522664, 522675, 522686, 522734, 522745, 522756, 522760, 522771, 522782, 522793, 522804, 522815, 522826, 522830, 522841, 522852, 522863, 522874, 522885, 522896, 522900, 522911, 522922, 522933, 522944, 522955, 522966, 522970, 522981, 523014, 523025, 523036, 523040, 523051, 523062, 523073, 523084, 523095, 523106, 523110, 523121, 523132, 523143, 523154, 523165, 523176, 523180, 523191, 523202, 523213, 523224, 523235, 523246, 523250, 523261, 523272, 523283, 523294, 523305, 523316, 523320, 523331, 523342, 523353, 523364, 523375, 523386, 523390, 523401, 523412, 523423, 523434, 523445, 523456, 523460, 523471, 523482, 523493, 523504, 523515, 523526, 523530, 523541, 523552, 523563, 523574, 523585, 523596, 523600, 523611, 523622, 523633, 523644, 523655, 523666, 523670, 523681, 523692, 523703, 523714, 523725, 523736, 523740, 523751, 523762, 523773, 523784, 523795, 523806, 523810, 523821, 523832, 523843, 523854, 523865, 523876, 523880, 523891, 523902, 523913, 523924, 523935, 523946, 523950, 523961, 523972, 523983, 523994, 524005, 524016, 524020, 524031, 524042, 524053, 524064, 524075, 524086, 524090, 524101, 643451, 643462, 653656, 653660, 770394, 770405, 770416, 770420 |
| **MUSCULOSKELETAL** | |
| Connective tissue disease | **ICD-10:** M05.0, M05.1, M05.2**,** M05.3, M05.8, M05.9, M06.0, M06.3, M06.9, M32, M33, M34, M35, M36.0, M36.8 |
| Arthritis (any type) | **ICD-10:** L40.5, M02.3, M05, M06, M08, M13.0, M13.1, M15-M19, M45, M46.1, M46.8, M46.9  **ATC**: L04AA13, L04AA24  **Medical procedure code:** 478030, 478041 |
| Gout or other crystal-induced arthropathy | **ICD-10:** M10, M11, M1A  **ATC:** M04A |
| Musculoskeletal problems | **ICD-10:** M02, M07, M12.0, M12.1, M12.2, M12.3, M12.4, M12.8, M12.9, M13, M14, M24.0, M24.3, M24.6, M24.7, M24.8, M24.9, M25, M36.1, M36.2, M36.3, M36.4, M45, M46.0, M46.1, M46.4, M46.8, M46.9, M47, M48, M49, M50, M51, M53, M54, M80, M81, M84.3, M84.4, M84.5, M84.6, Z87.31, Z87.39 |
| **NERVOUS SYSTEM** | |
| Dementia | **ICD-10:** A81.0, F01, F02, F03, F05, G30, G31.0, G31.83, G31.85  **ATC**: N06D |
| Cognitive deterioration (without dementia) | **ICD-10:** G31.1, G31.84, G31.89, G31.9, R41.81 |
| Parkinson’s disease | **ICD-10:** G20, G21, G23.1, G31.83, G31.85, G90.3  **ATC:** N04AB, N04AC, N04B |
| **PSYCHOLOGICAL AND BEHAVIOULAR** | |
| Depression | **ICD-10:** F06.31, F06.32, F30, F31, F32, F33, F34.1, F43.21, F43.23, F53.0  **ATC**: N06A |
| Anxiety | **ICD-10:** F40-F42 |
| Schizophrenia & paranoia | **ICD-10:** F06.0, F06.2, F20, F22, F23, F24, F28, F29 |
| **SKIN** | |
| Chronic skin ulcer | **ICD-10:** E08.621, E08.622, E09.621, E09.622, E10.621, E10.622, E11.621, E11.622, E13.621, E13.622, L89, L97, L98.4  **Medical procedure code:** 114074, 114085 |
| **VIRAL AND BACTERIAL INFECTIONS** | |
| HIV/AIDS | **ICD-10:** B20, B97.35, Z21  **ATC:** J05AE01 , J05AE03, J05AE04, J05AE05, J05AE07, J05AE08, J05AE09, J05AE10, J05AF01, J05AF02, J05AF03, J05AF04, J05AF06, J05AF09, J05AF11, J05AF12, J05AG, J05AJ, J05AR, J05AX07, J05AX09, J05AX29 |
| Skin and soft tissue infections | **ICD-10:** A06.7, A28.1, A31.1, A43.1, A46, A50.06, A51.3, A60.1, A63.0, A66.2, L00, L01, L02, L03, L04, L05, L08, L88  **Medical procedure codes:** 145552, 145563, 145574, 145585, 220253, 220264, 244650, 244661 |
| Mycosis^10^ | **ICD-10:** B35-B49  **ATC:** D01A, D01BA02 |
| **COMEDICATION USE (≤1 year before index date)^4^** | |
| Acetylsalicylic acid (low dose) | **ATC:** B01AC06 |
| Antacid | **ATC**: A02A |
| Clopidogrel | **ATC:** B01AC04 |
| CYP2C19 inhibitors | **ATC:** N03AX25 (cenobamaat), J05AG04 (etravirine), L01EJ02 (fedratinib), J02AC01, J01RA07 (fluconazole), N06AB03, N06CA03 (fluoxetine), N06AB08 (fluvoxamine), A16AX16 (givosiran), J04AM03, J04AC01, J04AC51, J04AM08, J04AM09, J04AM02, J04AM07, J04AM05, J04AM06, J04AM12, J04AM01, J04AM04 (isoniazide), N06AG02 (moclobemide), N06BA07 (modafinil), B01AC05 (ticlopidine), N03AX11, A08AA51 (topiramaat), J02AC03 (voriconazole) |
| Direct oral anticoagulants (DOAC) | **ATC:** B01AE, B01AF |
| NSAID | **ATC:** C08CA51, M01AA, M01AB, M01AC, M01AE, M01AG, M01AH, N02AJ08, N02AJ14, N02AJ19 |
| SSRI/SNRI | **ATC:** N06AB, N06AX16, N06AX21, N06CA03 |
| Use of short-acting bronchodilators (SABD) | **ATC:** R03AC02, R03AC03, R03AL01, R03AL02, R03BB01  Categorized as^11^:   - Appropriate (0-2 canisters/year) - Overuse (3-5 canisters/year) - Heavy overuse (>5 canisters/year)   With one standard canister defined as 200 doses |
| Vitamin K antagonist | **ATC:** B01AA |
| **CLINICAL RISK SCORES^4,5^** | |
| John Hopkins Claims-based Frailty Indicator^12-16^ | - **Impaired mobility**: beta coefficient 1.24 (definition mentioned above: ‘Impaired mobility’)  - **Depression**: beta coefficient 0.54 (definition mentioned above: ‘Depression’)  - **Congestive heart failure**: beta coefficient 0.50 (definition mentioned above: ‘Congestive heart failure’)  - **Parkinson’s disease**: beta coefficient 0.50 (definition mentioned above: ‘Parkinson’s disease’)  - **White race**: beta coefficient -0.49: not available  - **Arthritis (any type):** beta coefficient 0.43 (definition mentioned above: ‘Arthritis’)  - **Cognitive impairment**: beta coefficient 0.33 (combination of definitions mentioned above: ‘Dementia’ and  ‘Cognitive deterioration’)   - **ICD-10:** A81.0, F01, F02, F03, F05, G30, G31.0, G31.1, G31.83, G31.84, G31.85, G31.89, G31.9, R41.81 - **ATC:** N06D   - **Charlson comorbidity index** **(> 0)**: beta coefficient 0.31  - **Stroke**: beta coefficient 0.28 (definition mentioned above: ‘Stroke’)  - **Paranoia**: beta coefficient 0.24 (definition mentioned above: ‘Schizophrenia & paranoia’)  - **Chronic skin ulcer**: beta coefficient 0.23 (definition mentioned above: ‘Chronic skin ulcer’)  - **Pneumonia**: beta coefficient 0.21   - **ICD-10:** A01.03, A02.22, A37.01, A37.11, A37.81, A37.91, A50.04, A54.84, B01.2, B05.2, B06.81, B77.81, J09.X1, J09.X2, J09.X3, J10.0, J11.0, J12, J13, J14, J15, J16, J17, J18, J84.11, J84.2, J85.1, J95.851, Z87.01   - **Male sex**: beta coefficient -0.19  - **Skin and soft tissue infection**: beta coefficient 0.18 (definition mentioned above: ‘Skin and soft tissue  infection’)  - **Mycoses**: beta coefficient 0.14 (definition mentioned above: ‘Mycosis’)  - **Age (for every 1 year increase)**: beta coefficient 0.09  - **Admission in past 6 months**: beta coefficient 0.09  - **Gout or other crystal-induced arthropathy**: beta coefficient 0.08 (definition mentioned above: ‘Gout or  other crystal-induced arthropathy’)  - **Falls**: beta coefficient 0.08 (definition mentioned above: ‘History of falling’)  - **Musculoskeletal problems**: beta coefficient 0.05 (definition mentioned above: ‘Musculoskeletal problems’)  - **Urinary tract infection**: beta coefficient 0.05 (definition mentioned above: ‘Urinary tract infection) |
| Charlson Comorbidity Index^17-21^ | - **Myocardial infarction:** 1 point (definition mentioned above: ‘Myocardial infarction’)  - **Congestive heart failure**: 1 point (definition mentioned above: ‘Congestive heart failure’)  - **Peripheral vascular disease**: 1 point (definition mentioned above: ‘Peripheral vascular disease)  - **Cerebrovascular disease:** 1 point (definition mentioned above: ‘Cerebrovascular disease’)  - **Dementia**: 1 point (definition mentioned above: ‘Dementia’)  - **Connective tissue disease**: 1 point (definition mentioned above: ‘Connective tissue disease’)  - **Peptic ulcer disease**: 1 point (definition mentioned above: ‘Peptic ulcer disease’)  - **Mild liver disease**: 1 point (definition mentioned above: ‘Mild liver disease’)  - **Diabetes without chronic complications**: 1 point (definition mentioned above: ‘Diabetes’ and/or ‘Diabetes  without vascular complications’)  - **Diabetes with chronic complications**: 2 point (definition mentioned above: ‘Diabetes with vascular  complications)  - **Hemiplegia or paraplegia**: 2 points (definition mentioned above: ‘Hemiplegia/paraplegia)  - **Renal disease:** 2 points (definition mentioned above: ‘Chronic kidney disease’)  - **Any malignancy, including leukemia and lymphoma:** 2 points (definition mentioned above: ‘Any malignancy,  including leukemia and lymphoma’)  - **Moderate or severe liver disease:** 3 points (definition mentioned above: ‘Moderate or severe liver disease’)  - **Metastatic solid tumor:** 6 points (definition mentioned above: ‘Metastatic cancer’)  - **AIDS/HIV:** 6 points (definition mentioned above: ‘HIV’)  - **Age:**   - <50 years: 0 points - 50-59 years: 1 point - 60-69 years: 2 points - 70-79 years: 3 points - ≥80 years: 4 points   *The following comorbid conditions were mutually exclusive: diabetes with chronic complications and diabetes without chronic complications; mild liver disease and moderate or severe liver disease; and any malignancy and metastatic solid tumor*. Patients with diabetes without specific ICD-codes linked to diabetes with or without chronic complications were considered as patients with diabetes without chronic complications. |
| **OUTCOMES** | |
| Moderate exacerbations | Outpatient prescription fill for an oral corticosteroid (**ATC:** H02AB, except parental use (defined on package level)) and/or guideline-recommended antibiotics (**ATC:** 01CA, J01CR, J01DC, J01FA, J01MA, except continuous azithromycin therapy). Prescription fills needed to be separated by at least 14 days to be considered as separate events. Moderate exacerbations that led to hospital admission for a severe exacerbation within 14 days were classified only as hospitalized exacerbations, to avoid counting the same event twice. |
| Severe/hospitalized exacerbations | Hospital admissions or emergency department visits with a primary diagnosis code of asthma or (acute) COPD exacerbation, COPD with acute lower respiratory infection or asthma with status asthmaticus (**ICD-10:** J44.0, J44.1, J45.21, J45.22, J45.31, J45.32, J45.41, J45.42, J45.51, J45.52, J45.901 or J45.902) OR with a primary diagnosis of chronic lower respiratory disease (**ICD-10:** J40-J47) or respiratory failure (**ICD-10:** J96), combined with a secondary diagnosis code of asthma or (acute) COPD exacerbation, COPD with acute lower respiratory infection or asthma with status asthmaticus (**ICD-10:** J44.0, J44.1, J45.21, J45.22, J45.31, J45.32, J45.41, J45.42, J45.51, J45.52, J45.901 or J45.902). Hospitalizations with a concomitant pneumonia diagnosis were excluded (**ICD-10:** A01.03, A02.22, A37.01, A37.11, A37.81, A37.91, A50.04, A54.84, B01.2, B05.2, B06.81, B77.81, J09.X1, J09.X2, J09.X3, J10.0, J11.0, J12, J13, J14, J15, J16, J17, J18, J84.11, J84.2, J85.1, J95.851). |

Definition of in- and exclusion criteria, comorbidities, comedication, prescribing physician’s specialty and clinical risk scores based on ICD-coded hospital discharge diagnoses (ICD-9-CM until 2014, ICD-10-BE since 2015)^22^, medical procedure codes^23^, ATC-coded prescription claims^24^ and/or the last three digits of the physician’s identification code of the Belgian National Institute for health and Disability Insurance (RIZIV/INAMI)^25^.

*AIDS: acquired immunodeficiency syndrome; ATC: Anatomical Therapeutic Chemical Classification; COPD: Chronic obstructive pulmonary disease; DM: diabetes mellitus; GERD: gastroesophageal reflux disease; HIV = human immunodeficiency virus; ICD-9-CM: International Classification of Diseases (ICD) codes, 9^th^ revision, Clinical Modification; ICD-10-BE: International Classification of Diseases (ICD) codes, 10^th^ Revision, Belgian Modification; SABD = short-acting bronchodilators; SNRI = serotonin-norepinephrine reuptake inhibitor; SSRI = selective serotonin reuptake inhibitor.*

## e-Table 3: Baseline characteristics of the study population stratified by PPI dose category

|  | | Subgroups | | | | |
| --- | --- | --- | --- | --- | --- | --- |
|  |  | No PPI use (n= 516,048) | 1 - 28 DDDs  (n= 57,540) | 29 – 180 DDDs (n= 128,017) | 181 – 365 DDDs (n= 127,981) | > 365 DDDs  (n= 102,549) |
| **Patient characteristics** | |  |  |  |  |  |
| Female sex | | 262,694 (50.9%) | 32,824 (57.0%) | 72,983 (57.0%) | 73,658 (57.6%) | 59,814 (58.3%) |
| Age (years) | | 56.5 [40.0,70.1] | 58.2 [43.1,71.1] | 62.8 [49.8,74.7] | 69.8 [58.7,80.7] | 69.2 [58.8,79.6] |
|  | 18-50 years | 201,215 (39.0%) | 20,339 (35.3%) | 32,357 (25.3%) | 15,643 (12.2%) | 11,398 (11.1%) |
|  | ≥50-80 years | 253,399 (49.1%) | 29,967 (52.1%) | 74,397 (58.1%) | 78,341 (61.2%) | 66,525 (64.9%) |
|  | ≥80 years | 61,434 (11.9%) | 7,234 (12.6%) | 21,263 (16.6%) | 33,997 (26.6%) | 24,626 (24.0%) |
| Low SES | | 122,834 (23.8%) | 15,406 (26.8%) | 40,624 (31.7%) | 48,085 (37.6%) | 42,997 (41.9%) |
| (Ever-)smoking | | 88,637 (17.2%) | 15,724 (27.3%) | 36,967 (28.9%) | 39,879 (31.2%) | 35,141 (34.3%) |
| Weight | |  |  |  |  |  |
|  | Normal | 498,407 (96.6%) | 51,609 (89.7%) | 114,548 (89.5%) | 113,169 (88.4%) | 89,465 (87.2%) |
|  | Cachexia/underweight | 2,248 (0.4%) | 914 (1.6%) | 2,255 (1.8%) | 2,210 (1.7%) | 1,732 (1.7%) |
|  | Obesity/overweight | 15,393 (3.0%) | 5,017 (8.7%) | 11,214 (8.8%) | 12,602 (9.8%) | 11,352 (11.1%) |
| Exacerbation history | |  |  |  |  |  |
|  | No exacerbation | 214,800 (41.6%) | 16,591 (28.8%) | 36,320 (28.4%) | 33,848 (26.4%) | 25,375 (24.7%) |
|  | One moderate exacerbation | 146,699 (28.4%) | 16,735 (29.1%) | 35,496 (27.7%) | 34,424 (26.9%) | 25,899 (25.3%) |
|  | Two or more moderate or one severe exacerbation | 154,549 (29.9%) | 24,214 (42.1%) | 56,201 (43.9%) | 59,709 (46.7%) | 51,275 (50.0%) |
| Comorbidities | |  |  |  |  |  |
| Arthritis | | 4,761 (0.9%) | 2,414 (4.2%) | 4,635 (3.6%) | 5,429 (4.2%) | 4,785 (4.7%) |
| Bronchiectasis | | 875 (0.2%) | 294 (0.5%) | 634 (0.5%) | 849 (0.7%) | 784 (0.8%) |
| Cancer | | 16,933 (3.3%) | 4,676 (8.1%) | 11,237 (8.8%) | 11,955 (9.3%) | 9,862 (9.6%) |
| Cardiovascular comorbidity | | 128,291 (24.9%) | 20,636 (35.9%) | 53,742 (42.0%) | 68,368 (53.4%) | 57,862 (56.4%) |
| Depression/anxiety | | 91,194 (17.7%) | 14,112 (24.5%) | 38,136 (29.8%) | 48,540 (37.9%) | 43,453 (42.4%) |
| Diabetes mellitus | | 58,415 (11.3%) | 8,221 (14.3%) | 23,676 (18.5%) | 31,422 (24.6%) | 28,254 (27.6%) |
| Frailty | | 41,934 (8.1%) | 6,110 (10.6%) | 18,421 (14.4%) | 31,281 (24.4%) | 23,722 (23.1%) |
| GERD | | 1,278 (0.2%) | 851 (1.5%) | 5,841 (4.6%) | 8,051 (6.3%) | 7,966 (7.8%) |
| Sleep apnea | | 15,342 (3.0%) | 2,346 (4.1%) | 6,453 (5.0%) | 7,789 (6.1%) | 7,082 (6.9%) |
| Stomach ulcer | | 888 (0.2%) | 544 (0.9%) | 3,046 (2.4%) | 3,852 (3.0%) | 3,741 (3.6%) |
| Medication use | |  |  |  |  |  |
| Acetylsalicylic acid | | 82,258 (15.9%) | 12,006 (20.9%) | 33,019 (25.8%) | 44,529 (34.8%) | 37,642 (36.7%) |
| Antacid | | 16,224 (3.1%) | 2,760 (4.8%) | 8,735 (6.8%) | 11,737 (9.2%) | 10,605 (10.3%) |
| Clopidogrel | | 10,125 (2.0%) | 1,828 (3.2%) | 5,307 (4.1%) | 7,913 (6.2%) | 6,951 (6.8%) |
| DOAC | | 21,479 (4.2%) | 3,673 (6.4%) | 10,310 (8.1%) | 14,407 (11.3%) | 11,374 (11.1%) |
| NSAID | | 192,181 (37.2%) | 33,691 (58.6%) | 63,096 (49.3%) | 54,076 (42.3%) | 46,414 (45.3%) |
| SABD use | |  |  |  |  |  |
|  | Appropiate | 446,583 (86.5%) | 50,231 (87.3%) | 111,354 (87.0%) | 110,895 (86.6%) | 85,244 (83.1%) |
|  | Overuse | 39,806 (7.7%) | 4,126 (7.2%) | 9,116 (7.1%) | 9,139 (7.1%) | 8,645 (8.4%) |
|  | Heavy overuse | 29,659 (5.7%) | 3,183 (5.5%) | 7,547 (5.9%) | 7,947 (6.2%) | 8,660 (8.4%) |
| Vitamin K antagonist | | 9,914 (1.9%) | 1,250 (2.2%) | 3,584 (2.8%) | 5,465 (4.3%) | 4,653 (4.5%) |

*DDD: defined daily dose; DOAC: direct oral anticoagulants; PPI: proton pump inhibitor; SES: socio-economic status; GERD: gastroesophageal reflux disease; SABD: short-acting bronchodilator; NSAID: non-steroidal anti-inflammatory drug
SABD appropriate use: 0-2 canisters/year; SABD overuse: 3-5 canisters/year; SABD heavy overuse (>5 canisters/year).*

## e-Table 4: The number of patients and crude event rates for severe exacerbations with 95% CI per 100 person-years at risk for each PPI dose category

|  | Number of patients (%) | Severe exacerbation rate per 100 PY (95%CI) |
| --- | --- | --- |
| No PPI use | 516,048 (55.4%) | 0.91 (0.90-0.93) |
| Overall PPI use | 416,087 (44.6%) | 1.90 (1.87-1.92) |
| 1 - 28 DDDs | 57,540 (6.2%) | 1.50 (1.44-1.56) |
| 29 - 180 DDDs | 128,017 (13.7%) | 1.68 (1.64-1.72) |
| 181 - 365 DDDs | 127,981 (13.7%) | 1.99 (1.94-2.04) |
| >365 DDDs | 102,549 (11.0%) | 2.28 (2.23-2.34) |

*CI = confidence interval; PPI = proton pump inhibitor; PY = person-years*

# Supplemental figures

## e-Figure 1: Overview of the study design


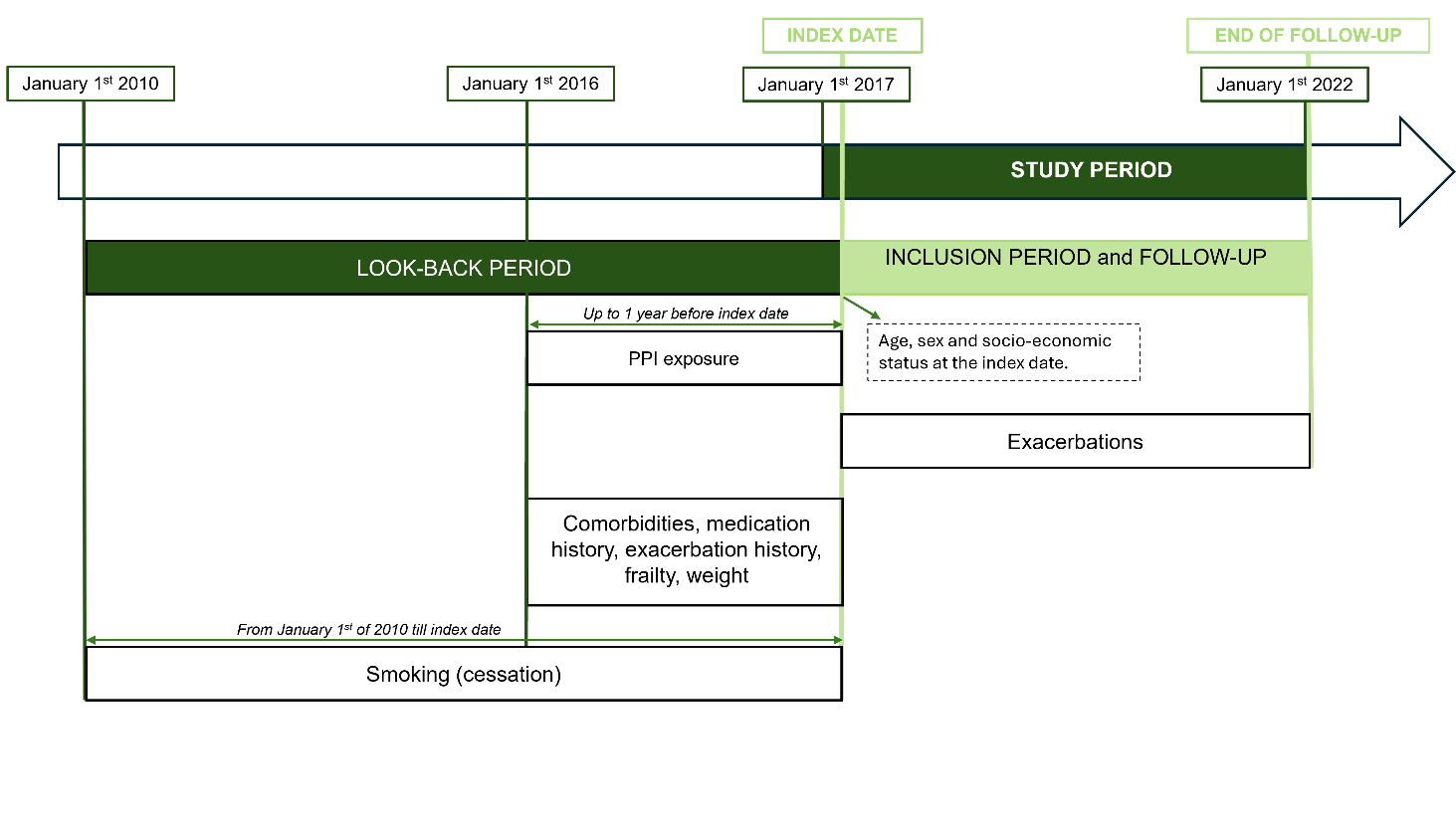


**Study period:** January 1^st^, 2017 – January 1^st^, 2022. **Follow-up:** Period between index date and end of follow-up. **End of follow-up:** Follow-up ended in case of the first occurrence of the investigated outcome (exacerbation), death, emigration, or end of the study period, whichever came first. **Look-back period:** Up to 1 year before index date (maximum until January 1^st^, 2016) for comorbidities, medication use, and health care resource use/costs and hospitalizations and from January 1^st^, 2010 until index date for smoking (cessation). **Index date:** The dispensing date of the second outpatient delivery of a drug for obstructive airway diseases (ATC R03) in one year (not necessarily a calendar year) during the study period to subjects ≥ 18 years with a membership of at least 1 year to a Belgian Health insurance fund. **Comorbidities:** Comorbidities were identified using specific ICD-coded diagnoses from the MHD, medical procedure codes from the IMA database and/or ATC-coded prescription claims from the IMA database in the year before the index date. **Medication use:** Medication dispensed up to 1 year before the index date.

*ATC: Anatomical Therapeutic Chemical Classification; IMA: InterMutualistic Agency; MHD: Minimal Hospital Dataset*

## e-Figure 2: Love plots

1. Covariate balance before and after IPTW: overall PPI use vs no PPI use


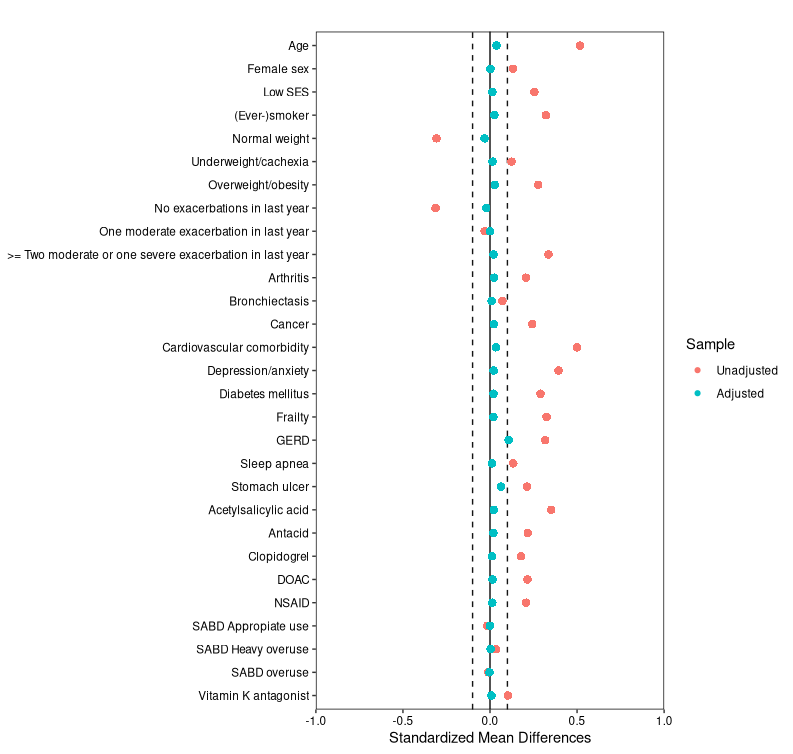


*DOAC: direct oral anticoagulants; PPI: proton pump inhibitor; SES: socio-economic status; GERD: gastroesophageal reflux disease; SABD: short-acting bronchodilator; NSAID: non-steroidal anti-inflammatory drug*

Because the Standardized Mean Difference (SMD) for GERD was 0.1068, which exceeded 0.1, the IPTW-weighted Cox analysis was adjusted for GERD.

1. Covariate balance before and after IPTW: 1-28 DDDs vs no PPI use


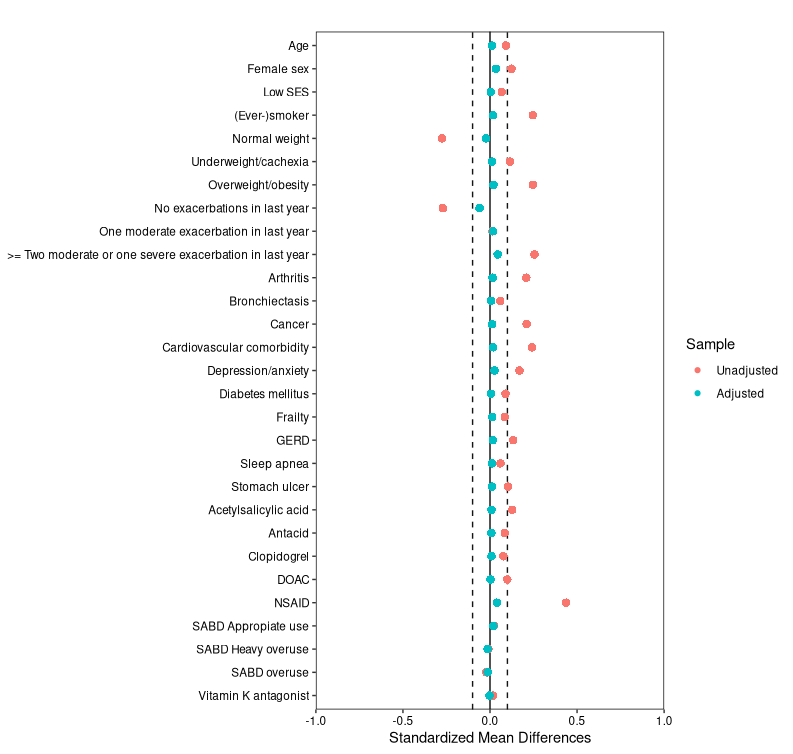


*DDD: defined daily dose; DOAC: direct oral anticoagulants; PPI: proton pump inhibitor; SES: socio-economic status; GERD: gastroesophageal reflux disease; SABD: short-acting bronchodilator; NSAID: non-steroidal anti-inflammatory drug*

1. Covariate balance before and after IPTW: 29-180 DDDs vs no PPI use


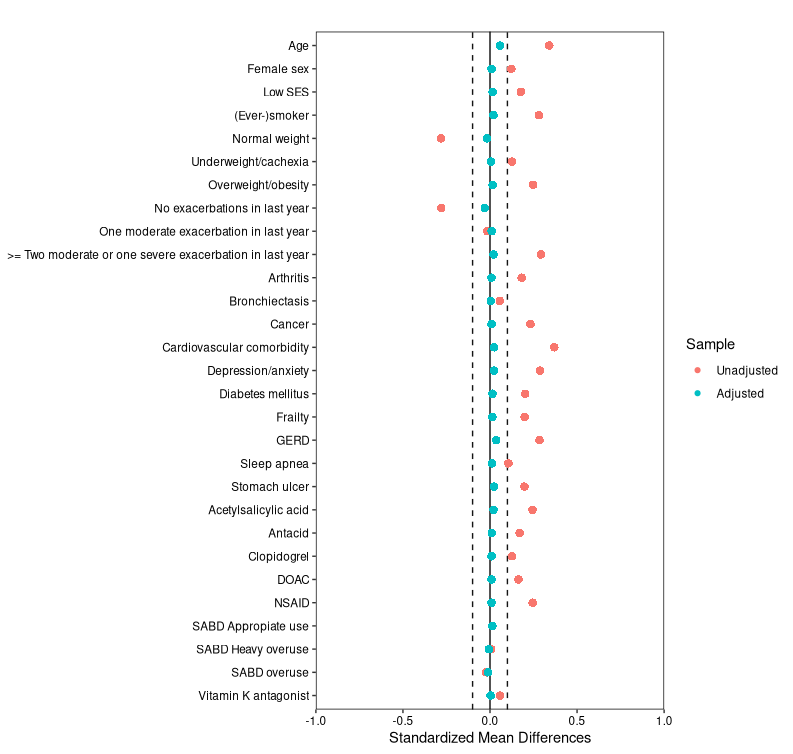


*DDD: defined daily dose; DOAC: direct oral anticoagulants; PPI: proton pump inhibitor; SES: socio-economic status; GERD: gastroesophageal reflux disease; SABD: short-acting bronchodilator; NSAID: non-steroidal anti-inflammatory drug*

1. Covariate balance before and after IPTW: 181-365 DDDs vs no PPI use


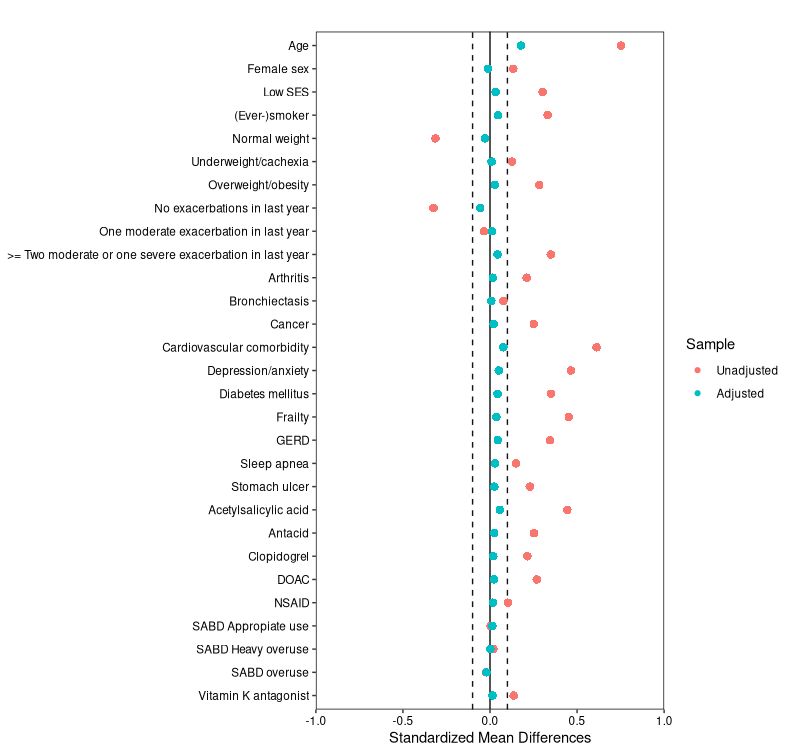


*DDD: defined daily dose; DOAC: direct oral anticoagulants; PPI: proton pump inhibitor; SES: socio-economic status; GERD: gastroesophageal reflux disease; SABD: short-acting bronchodilator; NSAID: non-steroidal anti-inflammatory drug*

Because the Standardized Mean Difference (SMD) for age was 0.1771, which exceeded 0.1, the IPTW-weighted Cox analysis was adjusted for age.

1. Covariate balance before and after IPTW: > 365 DDDs vs no PPI use


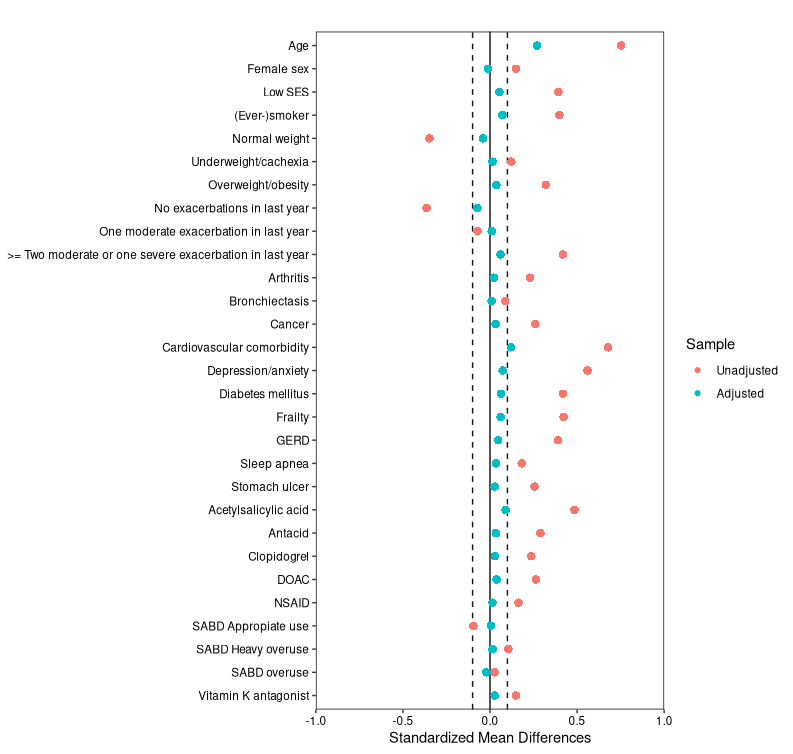


*DDD: defined daily dose; DOAC: direct oral anticoagulants; PPI: proton pump inhibitor; SES: socio-economic status; GERD: gastroesophageal reflux disease; SABD: short-acting bronchodilator; NSAID: non-steroidal anti-inflammatory drug*

Because the Standardized Mean Difference (SMD) for age and cardiovascular comorbidity exceeded 0.1 (0.2694 and 0.1202, respectively), the IPTW-weighted Cox analysis was adjusted for age and cardiovascular comorbidity.

## e-Figure 3: Flowchart of study population.


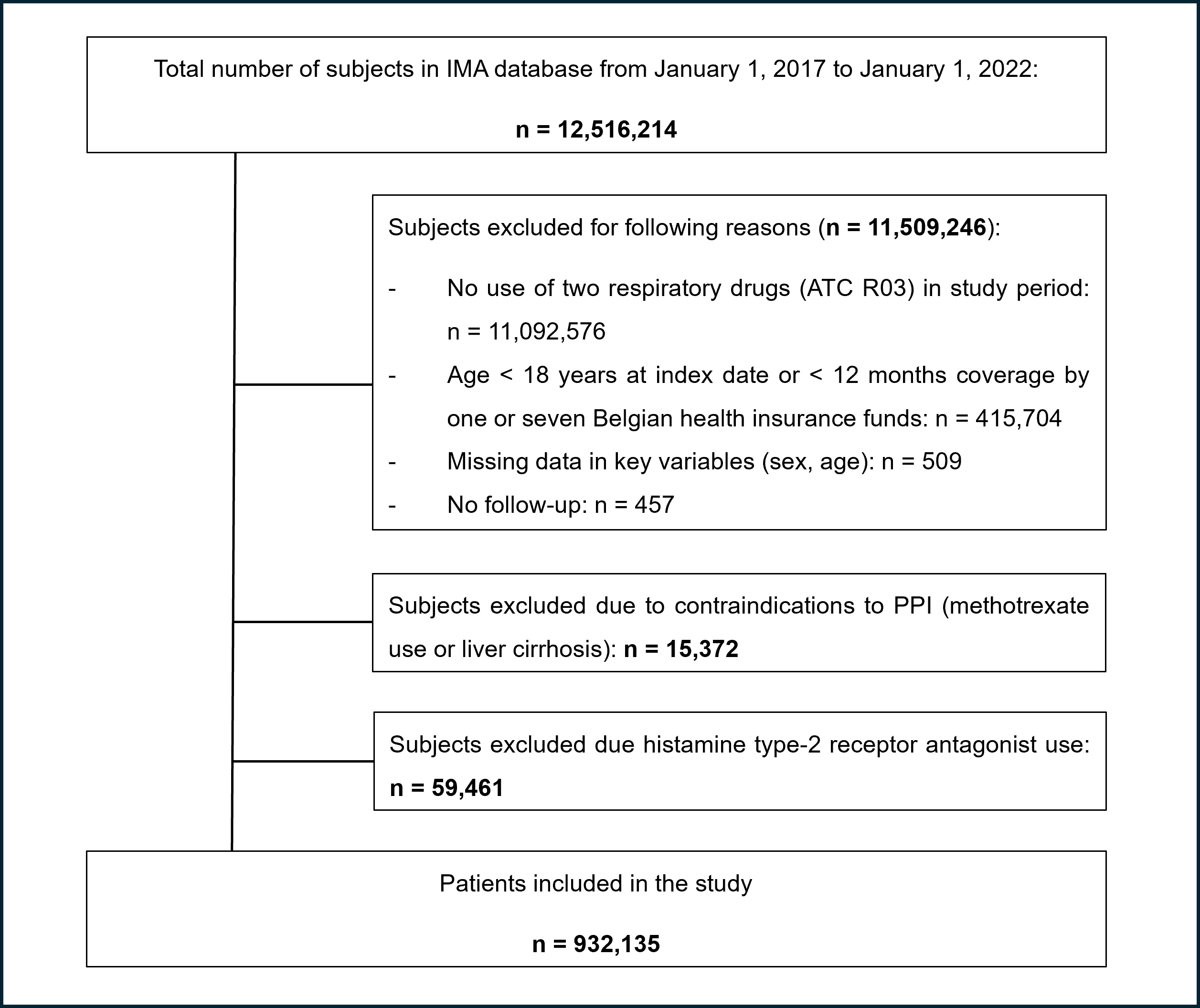


*ATC: Anatomical Therapeutic Chemical; IMA: InterMutualistic Agency; PPI: proton pump inhibitor*

## e-Figure 4: Cumulative incidence of exacerbations in COAD patients by PPI (dose)

1. Cumulative incidence of exacerbations by PPI use


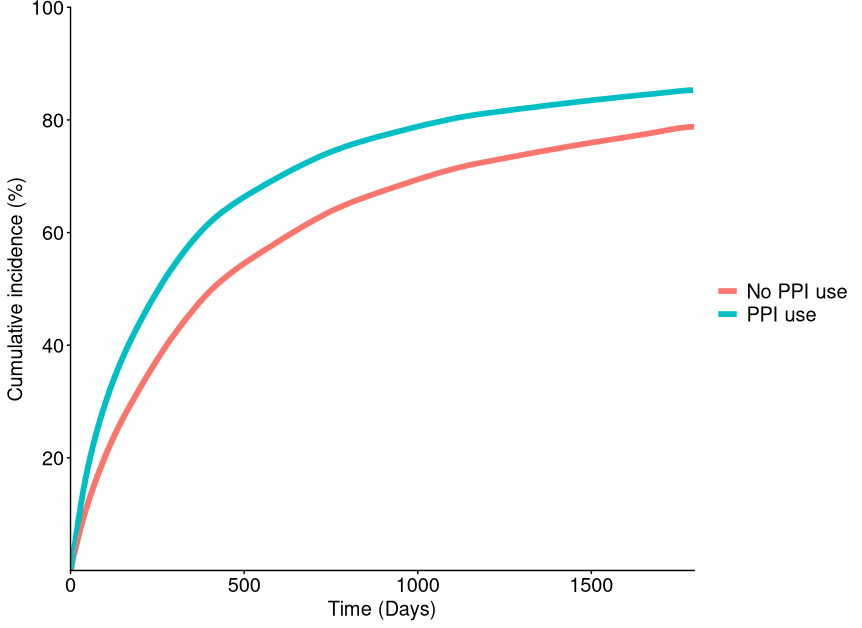


*PPI = proton pump inhibitor*

1. Cumulative incidence of exacerbations by PPI dose


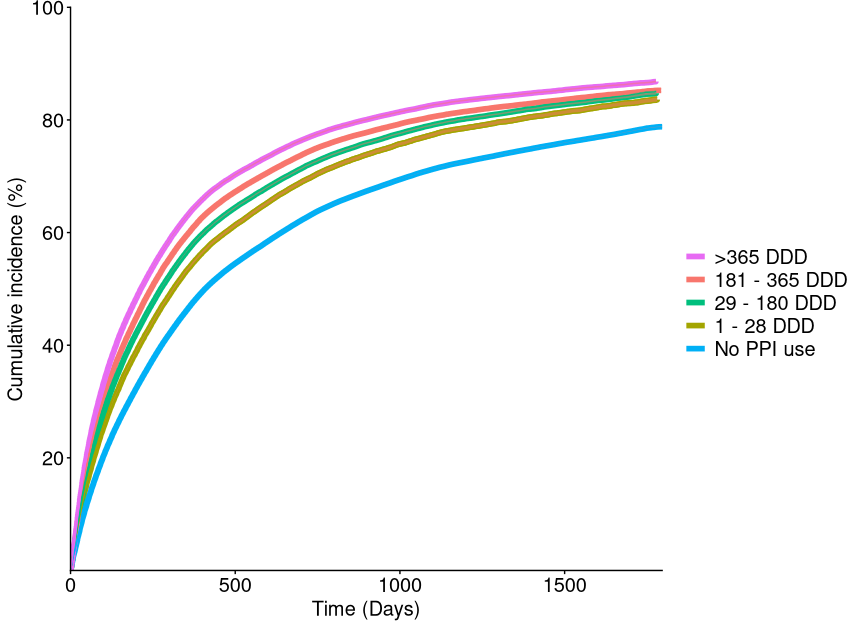


*DDD: defined daily dose; PPI = proton pump inhibitor*

## e-Figure 5: Cumulative incidence of severe exacerbations in COAD patients by PPI (dose)

1. Cumulative incidence of severe exacerbations by PPI use


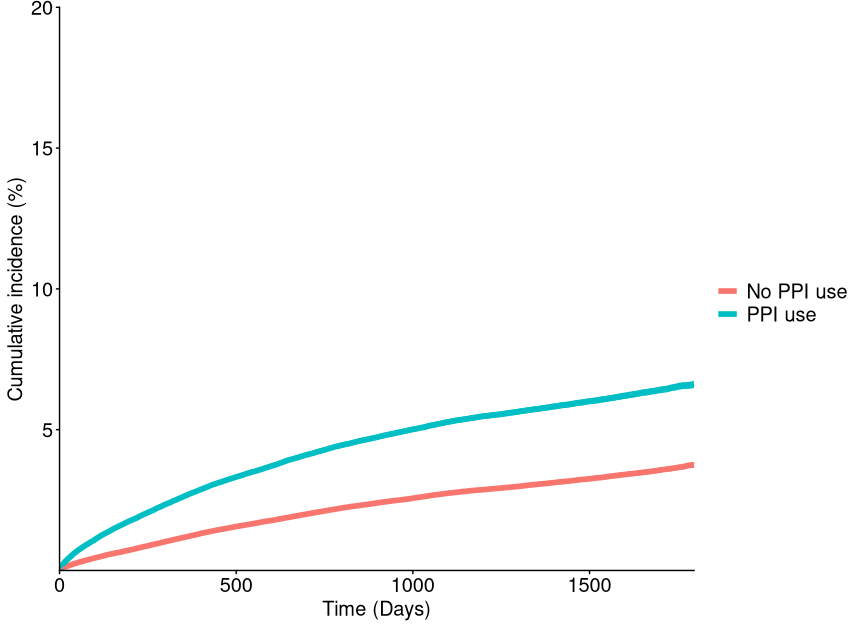


*PPI = proton pump inhibitor*

1. Cumulative incidence of severe exacerbations by PPI dose


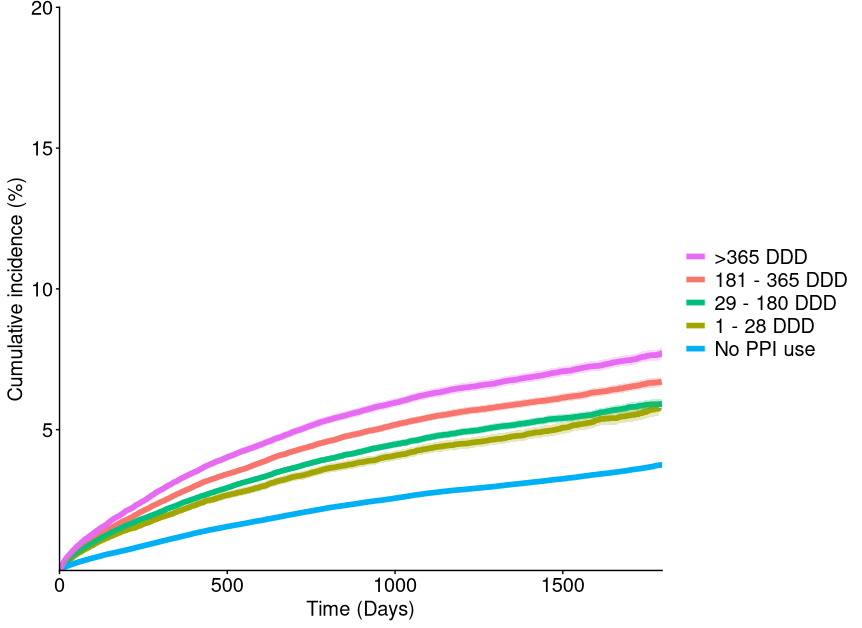


*DDD: defined daily dose; PPI = proton pump inhibitor*

## e-Figure 6: Weighted Cox regression analyses of the (dose-dependent) association between proton pump inhibitor use and exacerbations stratified by GERD diagnosis

1. Association of overall PPI use (vs. no use) and exacerbations stratified by GERD diagnosis


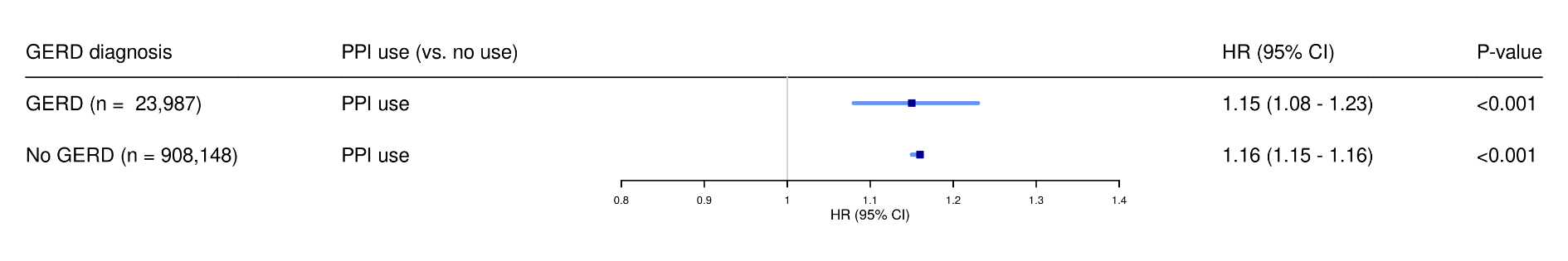

*CI: confidence interval; DDD: defined daily dose; GERD: gastroesophageal reflux disease; HR: hazard ratio; PPI: proton pump inhibitors*

1. Dose-dependent association of PPI use (vs. no use) and exacerbations stratified by GERD diagnosis

*
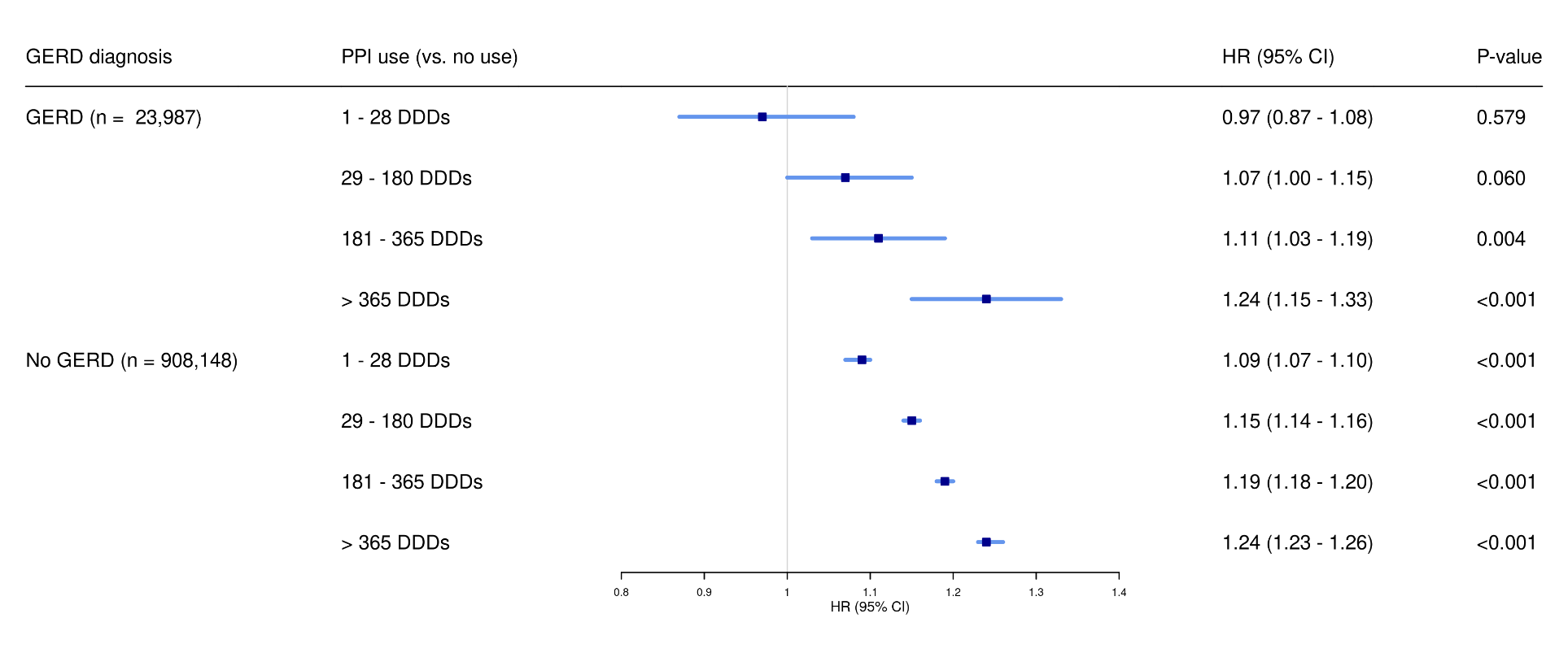

CI: confidence interval; DDD: defined daily dose; GERD: gastroesophageal reflux disease; HR: hazard ratio; PPI: proton pump inhibitors*

## e-Figure 7: Weighted Cox regression analyses of the dose-dependent association between proton pump inhibitor use and exacerbations stratified by age groups


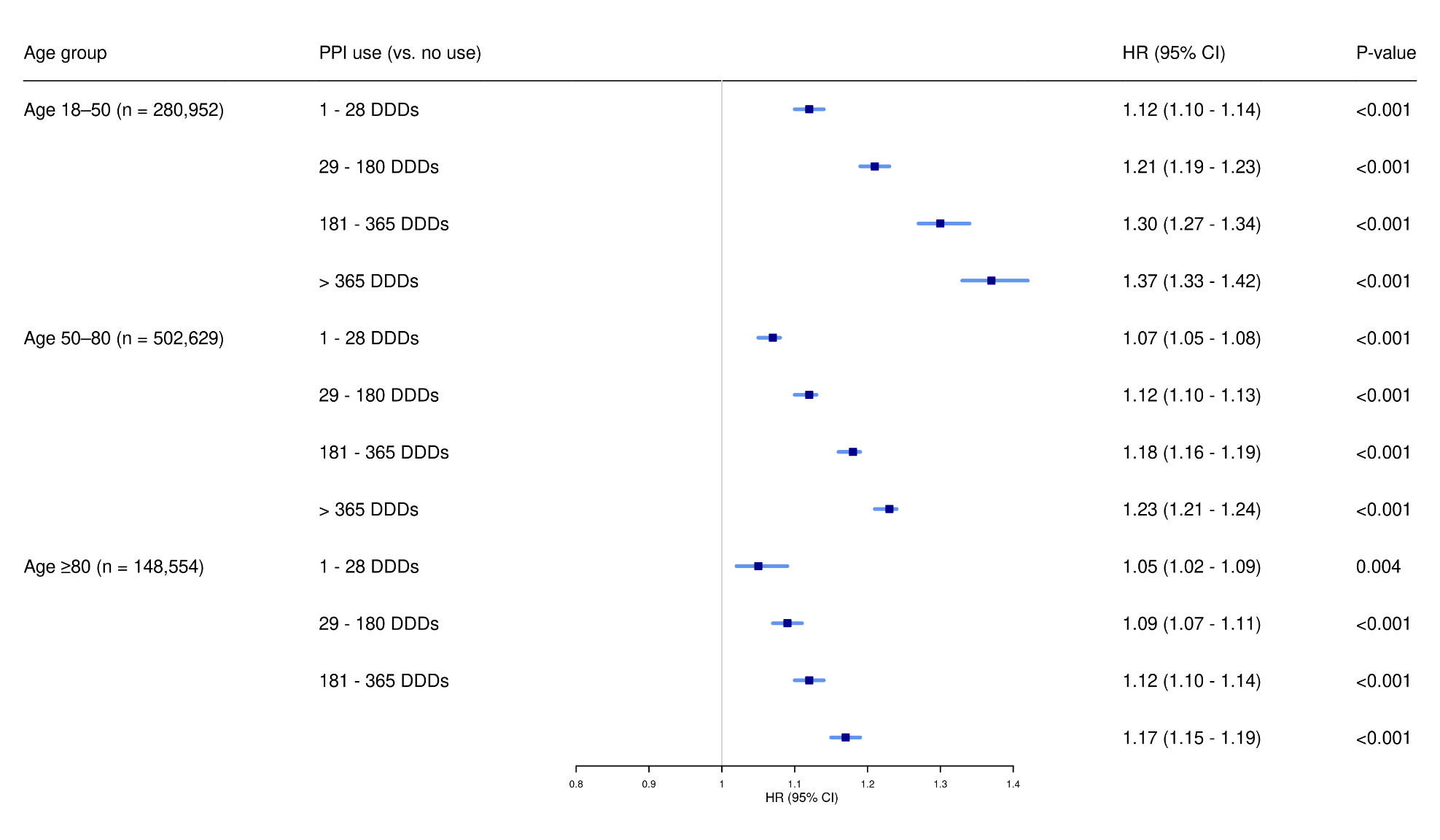


*CI: confidence interval; DDD: defined daily dose; GERD: gastroesophageal reflux disease; HR: hazard ratio; PPI: proton pump inhibitors*

## e-Figure 8: Weighted Cox regression analyses of the dose-dependent association between proton pump inhibitor use and exacerbations stratified by frailty status

1. Association of overall PPI use (vs. no use) and exacerbations stratified by frailty status

*
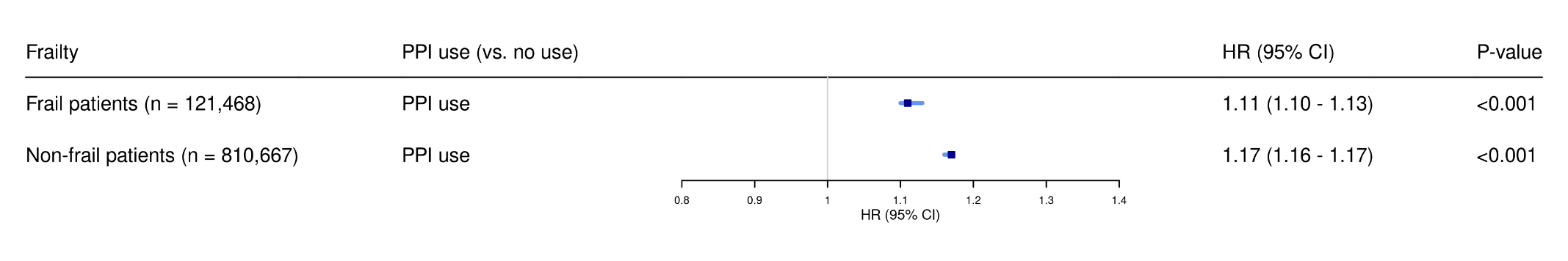
*

*CI: confidence interval; DDD: defined daily dose; GERD: gastroesophageal reflux disease; HR: hazard ratio; PPI: proton pump inhibitors*

1. Dose-dependent association of PPI use (vs. no use) and exacerbations stratified by frailty status

*
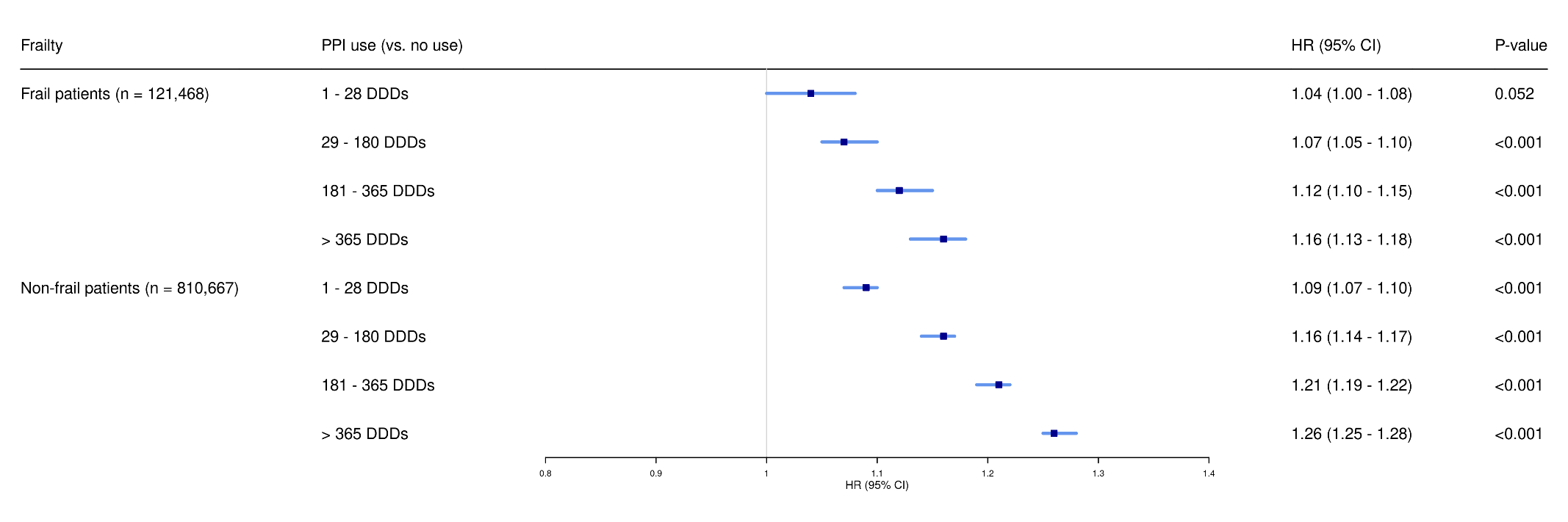
*

*CI: confidence interval; DDD: defined daily dose; GERD: gastroesophageal reflux disease; HR: hazard ratio; PPI: proton pump inhibitors*

## e-Figure 9: Cox regression analyses of the association between actual proton pump inhibitor use on index date and exacerbations, with censored follow-up (n = 786,721)


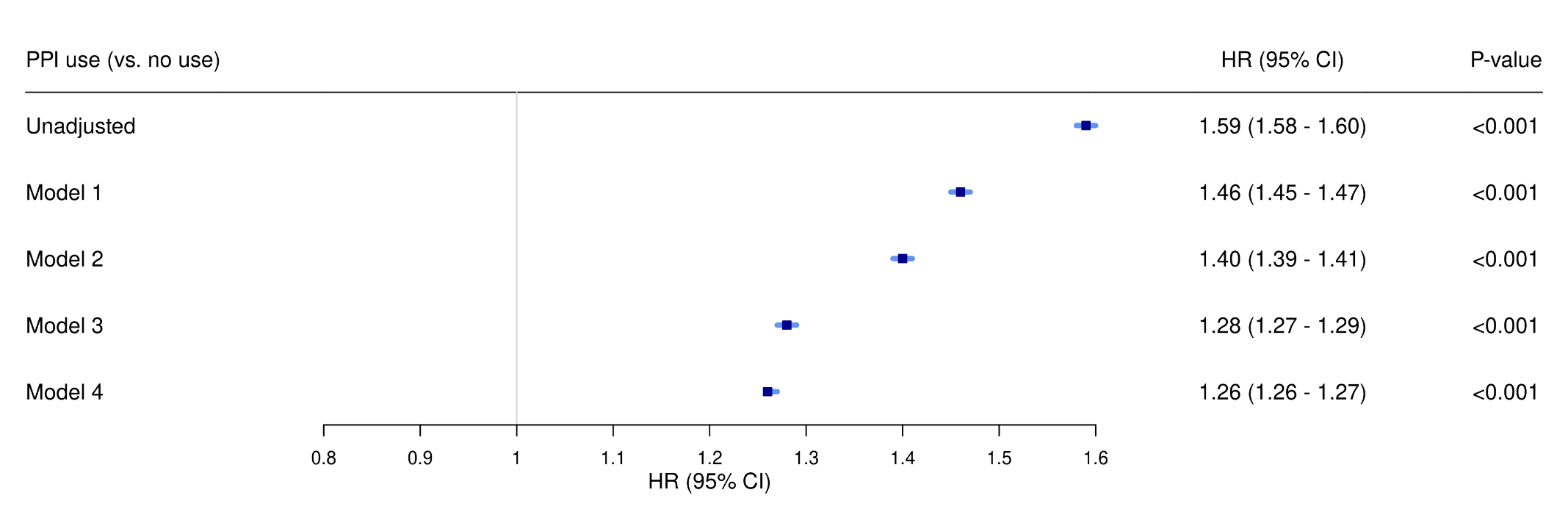


Model 1: adjusted for age and sex
Model 2: model 1 + smoking history, socio-economic status
Model 3: model 2 + exacerbation history and short-acting bronchodilator use
Model 4: model 3 + frailty, age-adjusted Charlson-Comorbidity Index and GERD

Patients who used a PPI during baseline but not on index date were excluded (n = 145.414).

*CI: confidence interval; HR: hazard ratio*

## e-Figure 10: Weighted Cox regression analyses of the dose-dependent association between proton pump inhibitor use and exacerbations, with short-term PPI use divided (≤ 1 week vs. > 1 week) (n = 932,135)


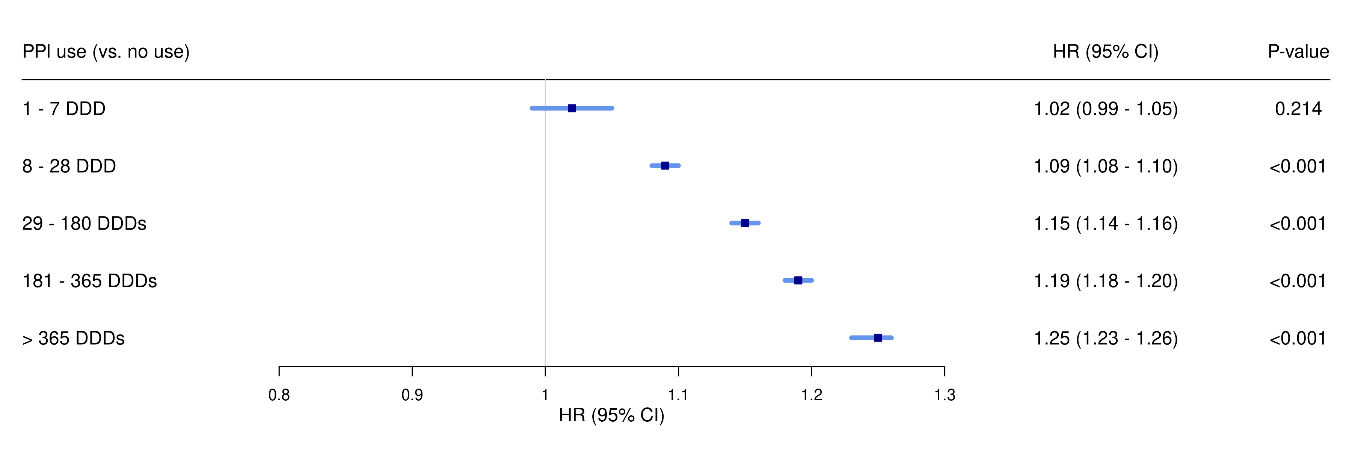


*CI = confidence interval; DDD = defined daily dose; HR = hazard ratio; PPI = proton pump inhibitors*

## e-Figure 11: In PPI users on index date, Cox regression analyses of the association between simultaneous CYP2C19 inhibitor use and exacerbations, with censored follow-up (n = 244,318)


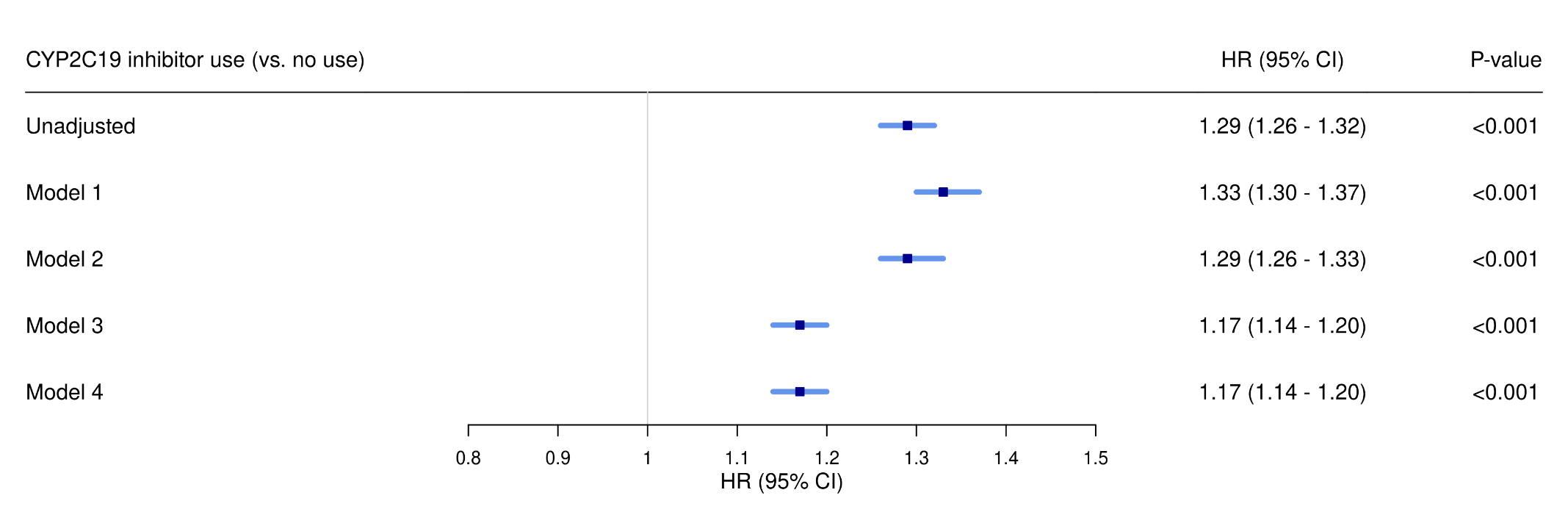


Model 1: adjusted for age and sex
Model 2: model 1 + smoking history, socio-economic status
Model 3: model 2 + exacerbation history and short-acting bronchodilator use
Model 4: model 3 + frailty, age-adjusted Charlson-Comorbidity Index and GERD

Patients who used a CYP2C19 inhibitor (n = 26.355) or PPI during baseline (n = 145.414) but not on index date were excluded.

*CI: confidence interval; HR: hazard ratio; GERD: gastroesophageal reflux disease*

## e-Figure 12: Cox regression analyses of the association between proton pump inhibitor use and severe exacerbations (n = 932,135)


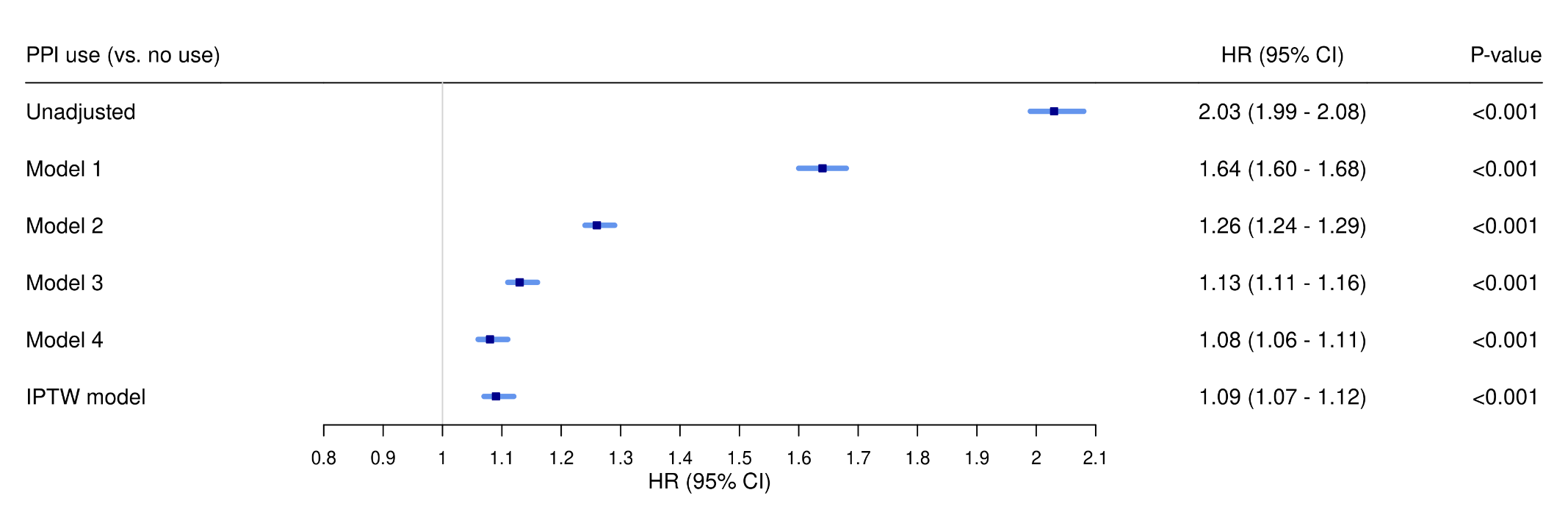


Model 1: adjusted for age and sex
Model 2: model 1 + smoking history, socio-economic status
Model 3: model 2 + exacerbation history and short-acting bronchodilator use
Model 4: model 3 + frailty, age-adjusted Charlson-Comorbidity Index and GERD

*CI = confidence interval; HR = hazard ratio*

# References

1. Maetens A, De Schreye R, Faes K, et al. Using linked administrative and disease-specific databases to study end-of-life care on a population level. *BMC Palliat Care*. Oct 18 2016;15(1):86. doi:10.1186/s12904-016-0159-7

2. IMA/AIM. InterMutualistic Agency (IMA/AIM). Accessed July 12, 2024. <https://ima-aim.be/>

3. MZG. The minimal hospital dataset. Accessed July 12, 2024. <https://www.health.belgium.be/en/node/23607>

4. WHO Collaborating Centre for Drug Statistics Methodology. Available from: <https://www.whocc.no/>. Accessed 25 November 2021.

5. The International Classification of Diseases (ICD), Clinical Modification. Available from: <https://www.cdc.gov/nchs/icd/index.htm>. Accessed 25 November 2021.

6. RIZIV/INAMI (Rijksinstituut voor ziekte- en invaliditeitsverzekering/Institut national d'assurance maladie-invalidité) medical procedure codes for claims of ambulatory and hospital care. Available from: <https://www.riziv.fgov.be/nl/nomenclatuur/Paginas/default.aspx> (in Dutch/French). Accessed 25 November 2021.

7. Lorenzoni G, Baldi I, Soattin M, Gregori D, Buja A. A Systematic Review of Case-Identification Algorithms Based on Italian Healthcare Administrative Databases for Three Relevant Diseases of the Cardiovascular System: Hypertension, Heart Failure, and Congenital Heart Diseases. *Epidemiol Prev*. Jul-Aug 2019;43(4 Suppl 2):51-61. doi:10.19191/ep19.4.S2.P051.092

8. Chakhtoura M, Haber R, Ghezzawi M, Rhayem C, Tcheroyan R, Mantzoros CS. Pharmacotherapy of obesity: an update on the available medications and drugs under investigation. *EClinicalMedicine*. Apr 2023;58:101882. doi:10.1016/j.eclinm.2023.101882

9. Kranz J, Schmidt S, Lebert C, et al. The 2017 Update of the German Clinical Guideline on Epidemiology, Diagnostics, Therapy, Prevention, and Management of Uncomplicated Urinary Tract Infections in Adult Patients. Part II: Therapy and Prevention. *Urol Int*. 2018;100(3):271-278. doi:10.1159/000487645

10. Thompson GR, 3rd, Le T, Chindamporn A, et al. Global guideline for the diagnosis and management of the endemic mycoses: an initiative of the European Confederation of Medical Mycology in cooperation with the International Society for Human and Animal Mycology. *Lancet Infect Dis*. Dec 2021;21(12):e364-e374. doi:10.1016/s1473-3099(21)00191-2

11. de Las Vecillas L, Quirce S. Landscape of short-acting beta-agonists (SABA) overuse in Europe. *Clin Exp Allergy*. Feb 2023;53(2):132-144. doi:10.1111/cea.14250

12. Kundi H, Coskun N, Yesiltepe M. Association of entirely claims-based frailty indices with long-term outcomes in patients with acute myocardial infarction, heart failure, or pneumonia: a nationwide cohort study in Turkey. *The Lancet Regional Health -Europe*. Nov 2021;10:100183. doi:10.1016/j.lanepe.2021.100183

13. Segal JB, Chang HY, Du Y, Walston JD, Carlson MC, Varadhan R. Development of a Claims-based Frailty Indicator Anchored to a Well-established Frailty Phenotype. *Med Care*. Jul 2017;55(7):716-722. doi:10.1097/mlr.0000000000000729

14. Segal JB, Huang J, Roth DL, Varadhan R. External validation of the claims-based frailty index in the national health and aging trends study cohort. *Am J Epidemiol*. 2017;186(6):745-747.

15. Grymonprez M, Petrovic M, De Backer TL, Steurbaut S, Lahousse L. Impact of frailty on the effectiveness and safety of non-vitamin K antagonist oral anticoagulants (NOACs) in patients with atrial fibrillation: a nationwide cohort study. *Eur Heart J Qual Care Clin Outcomes*. Jan 12 2024;10(1):55-65. doi:10.1093/ehjqcco/qcad019

16. Le Pogam M-A, Seematter-Bagnoud L, Niemi T, et al. Development and validation of a knowledge-based score to predict Fried's frailty phenotype across multiple settings using one-year hospital discharge data: The electronic frailty score. *eClinicalMedicine*. 2022;44:101260. doi:10.1016/j.eclinm.2021.101260

17. Quan H, Sundararajan V, Halfon P, et al. Coding algorithms for defining comorbidities in ICD-9-CM and ICD-10 administrative data. *Med Care*. Nov 2005;43(11):1130-9. doi:10.1097/01.mlr.0000182534.19832.83

18. Sundararajan V, Henderson T, Perry C, Muggivan A, Quan H, Ghali WA. New ICD-10 version of the Charlson comorbidity index predicted in-hospital mortality. *J Clin Epidemiol*. Dec 2004;57(12):1288-94. doi:10.1016/j.jclinepi.2004.03.012

19. Charlson M, Szatrowski TP, Peterson J, Gold J. Validation of a combined comorbidity index. *J Clin Epidemiol*. Nov 1994;47(11):1245-51. doi:10.1016/0895-4356(94)90129-5

20. Grymonprez M, De Backer TL, Bertels X, Steurbaut S, Lahousse L. Long-term comparative effectiveness and safety of dabigatran, rivaroxaban, apixaban and edoxaban in patients with atrial fibrillation: A nationwide cohort study. *Front Pharmacol*. 2023;14:1125576. doi:10.3389/fphar.2023.1125576

21. Yang CC, Fong Y, Lin LC, et al. The age-adjusted Charlson comorbidity index is a better predictor of survival in operated lung cancer patients than the Charlson and Elixhauser comorbidity indices. *Eur J Cardiothorac Surg*. Jan 1 2018;53(1):235-240. doi:10.1093/ejcts/ezx215

22. The International Classification of Diseases (ICD), Clinical Modification. Accessed 1 February, 2024. <https://www.cdc.gov/nchs/icd/index.htm>

23. RIZIV/INAMI (Rijksinstituut voor ziekte- en invaliditeitsverzekering/Institut national d'assurance maladie-invalidité) medical procedure codes for claims of ambulatory and hospital care. Accessed 1 February, 2024. <https://www.riziv.fgov.be/nl/nomenclatuur/Paginas/default.aspx>

24. WHO Collaborating Centre for Drug Statistics Methodology. Accessed 1 February, 2024. <https://www.whocc.no/>

25. RIZIV/INAMI (Rijksinstituut voor ziekte- en invaliditeitsverzekering/Institut national d'assurance maladie-invalidité) physician’s speciality codes. Accessed 18 June, 2024. <https://www.riziv.fgov.be/nl/professionals/info-voor-allen/bevoegdheidscodes->
